# One-Step Construction of 1,3,4-Oxadiazoles with Anticancer Activity from Tertiary Amines via a Sequential Copper(I)-Catalyzed Oxidative Ugi/aza-Wittig Reaction

**DOI:** 10.3390/molecules29061253

**Published:** 2024-03-12

**Authors:** Mei Sun, Nong-Qi Mao, Sheng-Long Wang, Xin-Ming Han, Gang Yao, Ping Xue, Chong-Yang Zeng, Yu-Ting Liu, Kai Chen, Xiao-Qing Gao, Jun Xiong

**Affiliations:** 1 Anhui Key Laboratory of Low Temperature Co-Fired Materials, School of Chemistry and Material Engineering, Huainan Normal University, Huainan 232038, China; hssm202106@163.com (M.S.); cyzeng1028@126.com (C.-Y.Z.); ytliu2003@163.com (Y.-T.L.); kaichen8488@163.com (K.C.); xqgao2003@163.com (X.-Q.G.); 2 School of Pharmacy, Xianning Medical College, Hubei University of Science and Technology, Xianning 437100, China; maoyor@163.com (N.-Q.M.); wangsl202403@163.com (S.-L.W.); han360a@163.com (X.-M.H.); yyyyygcn@126.com (G.Y.); pingxue@hbust.edu.cn (P.X.)

**Keywords:** 1,3,4-oxadiazole, Cu-catalyzed, C-H functionalization, oxidative Ugi reaction, aza-Wittig reaction, anticancer

## Abstract

An unparalleled copper(I)-catalyzed synthesis of 1,3,4-oxadiazoles from tertiary amines in one step has been described. The one-pot reactions involving (N-isocyanimine)triphenylphosphorane, tertiary amines, and carboxylic acids resulted in the formation of 1,3,4-oxadiazoles in moderate to good yields through a consecutive oxidative Ugi/aza-Wittig reaction, enabling the direct functionalization of sp^3^ C-H bonds adjacent to the nitrogen atom. This method offered several notable advantages, including ligands-free, exceptional productivity and a high functional group tolerance. The preliminary biological evaluation demonstrated that compound **4f** inhibited hepatoma cells efficiently, suggesting potentially broad applications of the approach for synthesis and medicinal chemistry.

## 1. Introduction

**Figure 1:**
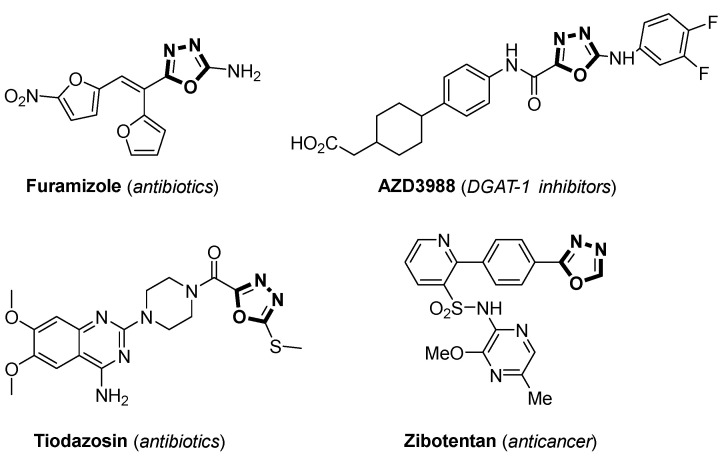
Representative biologically active 1,3,4-oxadiazole compounds.

Compounds containing 1,3,4-oxadiazoles have diverse applications in medicinal chemistry and material science [1,2,3,4,5,6]. These derivatives occupy an important position in heterocyclic compounds due to their wide range of biological and pharmacological properties, like antibiotic [7,8], antiproliferative [9], anticancer [10], antibacterial [11], and anti-inflammatory (Figure 1) [12]. Consequently, significant effort has been dedicated to synthesizing disubstituted 1,3,4-oxadiazoles [13,14,15,16]. In 2015, Wang revealed a palladium-catalyzed aminocarbonylation process employing chloroform as the carbon monoxide source to produce 1,3,4-oxadiazoles [17]. Subsequently, Ji and coworkers described a copper-catalyzed direct annulation of hydrazides for the synthesis of 1,3,4-oxadiazoles [18]. Recently, N-isocyaniminotriphenylphosphorane (NIITP), aryl iodides, and carboxylic acids have all be utilized to obtain 2,5-disubstituted 1,3,4-oxadiazoles by a one-pot synthesis–functionalization method described by Dixon [19]. Although some pathways are effective, they suffered from multi-step reactions, harsh conditions, and poor tolerance of the functional groups. Therefore, the development of a convenient construction of the variable 1,3,4-oxadiazole is still a fascinating theme.

**Scheme 1:**
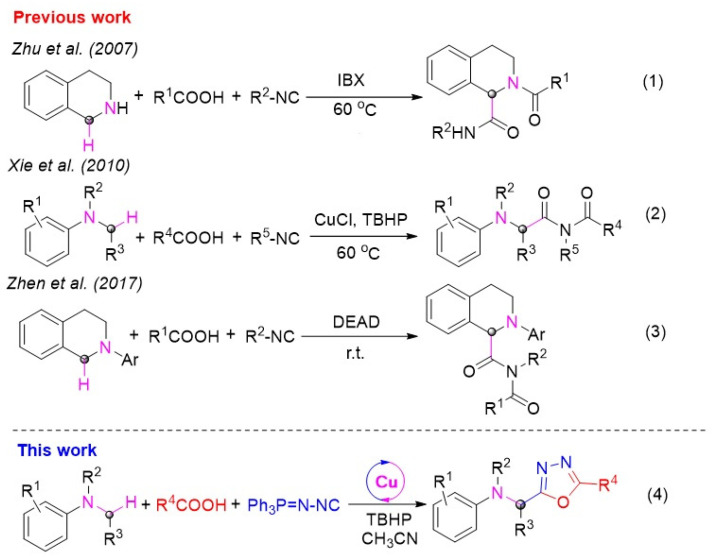
C(sp^3^)-H Bond Direct Functionalization of Tertiary Amines via Oxidative Ugi reaction [37,38,39].

In contrast to traditional organic synthesis, the process of functionalizing C(sp^3^)-H bonds through C-H bond activation has garnered considerable attention [20]. This is primarily due to its atom-economical nature and the elimination of laborious synthetic steps [21,22,23,24]. Notably, over the last decade, extensive research has been dedicated to the functionalization of sp^3^ C-H bonds via transition metal-catalyzed tertiary amine oxidation, leading to the formation of various chemical bonds such as C-C, C-P, and C-N linkages [25,26]. However, it is worth noting the scarcity of documented instances involving the direct functionalization of tertiary amines, concomitant with the grafting of C-H bonds onto an in situ-generated heterocyclic framework. Isocyanide-based multicomponent reactions (IMCRs) have demonstrated a lot of promise in heterocyclic synthesis in recent years [27,28,29,30]. As the demand for synthetic chemistry in terms of atomic economy and high efficiency [31], IMCRs have gradually developed from the original Ugi reaction [32,33] to an oxidative Ugi reaction [34] through the process of the amine component’s in situ oxidation to the imine ion. Among them, many elegant works on oxidative Ugi reaction have been reported which involves direct functionalization of the α-carbon atom of amines [35,36]. For instance, the IBX-mediated oxidative Ugi reaction, which involves sp^3^ C-H direct functionalization of tetrahydroisoquinolines, was first reported by Zhu’s lab in 2007 (Scheme 1, Equation (1)) [37]. Subsequently, the formation of α-amino imides from tertiary amines through a copper-catalyzed Ugi-type three-component reaction was initially described by Xie (Scheme 1, Equation (2)) [38]. Alternatively, DEAD-induced oxidative Ugi reactions have been reported by Zhen’s group (Scheme 1, Equation (3)) [39]. However, oxidative Ugi reactions which employ different oxidants, such as metal catalysts [40], diethyl azodicarboxylate (DEAD) [41], and photoredox catalysts [42], could be incompatible with the other reactions. As a result, the oxidative Ugi reaction of tertiary amines has grown into a more interesting and challenging field. However, to the best of our knowledge, the metal-catalyzed C(sp^3^)-H bond functionalization of tertiary amines with one-step construction of heterocyclic skeleton has not been reported. Owing to our continuing interest in the synthesis of heterocycle by employing domino IMCRs [43,44,45,46,47,48], we wish to develop a novel one-step construction of 1,3,4-oxadiazoles using the copper-catalyzed oxidative Ugi/aza-Wittig reaction (Scheme 1, Equation (4)).

## 2. Results and Discussion

We selected *N*-isocyanimine triphenylphosphorane **1**, 4-bromo-*N*, *N*-dimethylaniline **2a**, and 4-chlorobenzoic acid **3a** as model substrates for the consecutive copper(I)-catalyzed oxidative Ugi/aza-Wittig reaction to optimize the reaction conditions (Table 1). The intended product **4a** was obtained with a 79% yield by executing a three-component reaction using **1**, **2a**, and **3a** in the presence of CuCl/TBHP in CH_3_CN at 60 °C under dry N_2_ for 12 h (Table 1, entry 1). Nevertheless, reduced yields of **4a** ranging from 65% to 72% were achieved when the copper catalyst was replaced by CuBr_2_, CuI, or CuBr (Table 1, entries 2–4). Notably, the absence of a ligand in the reaction conditions of entry 1 did not have a detrimental effect on the reaction, as the desired product **4a** was successfully obtained in an 81% isolated yield (Table 1, entry 5). Remarkably, the reaction yield decreased in the presence of an air environment, resulting in a 66% yield (Table 1, entry 6). Switching the solvent to MeOH, THF, CH_2_Cl_2_, 1,4-dioxane, or PhMe (Table 1, entries 7–11) led to reduced reaction yields ranging from 24% to 69%. Furthermore, screening alternative peroxides such as BOP, DTBP, or TBPB did not yield satisfactory results (Table 1, entries 12–14, yields 38–65%). Subsequently, we investigated the influence of reaction temperature. Performing the reaction with CuCl/TBHP in CH_3_CN at 25 °C, 40 °C, or 80 °C (Table 1, entries 15–17) only resulted in a maximum reaction yield of 69%. Additionally, an attempt was made to reduce the catalyst loading to 5%, which led to a decrease in yield to 69% (Table 1, entry 18). When the reaction time was extended to 24 h, there was no significant change in yield (Table 1, entry 19). Upon transitioning from conventional heating to microwave heating, the yield unexpectedly decreased (Table 1, entry 20). The reaction conditions outlined in entry 5 of Table 1 were found to be optimal for the synthesis of **4a**, yielding 81%.

**Scheme 2:**
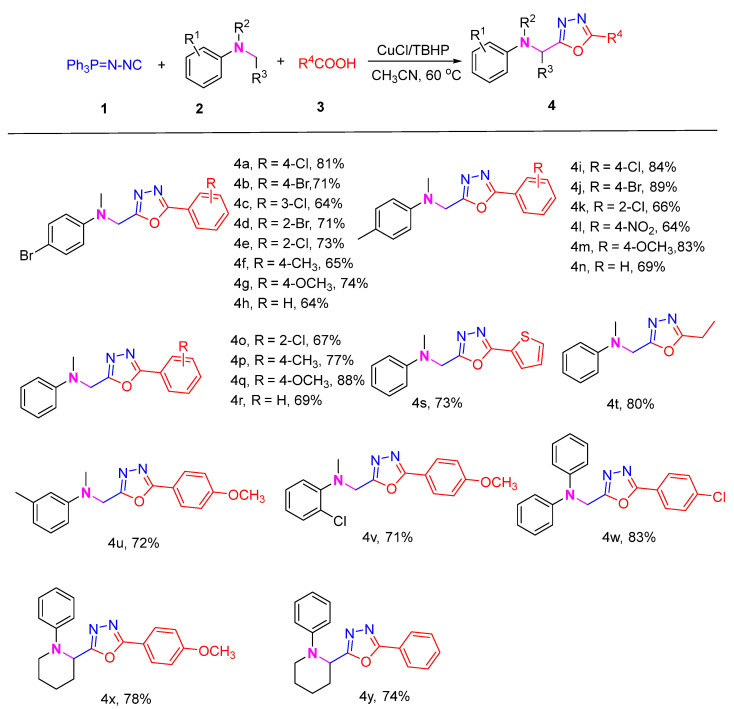
One-step preparation of 1,3,4-oxadiazoles **4**. Reaction conditions: **1** (1.0 mmol), **2** (2.0 mmol), **3** (1.5 mmol), TBHP (in decane, 1.5 mmol), CuCl (0.1 mmol), 4Å MS (300 mg), CH_3_CN (5 mL), N_2_, 60 °C, 12 h.

To ascertain the compatibility of this multicomponent reaction, various carboxylic acids **3** including halogen-substituted (R = 4-Br, 4-Cl, 3-Cl, 2-Br, 2-Cl), benzoic acids, smoothly reacted with *N*, *N*-dimethylanilines **2** and *N*-isocyaniminotriphenylphosphorane **1** (as demonstrated in Scheme 2) resulting in the generation of the corresponding 1,3,4-oxadiazoles **4**, yielding moderate to commendable proportions (Scheme 2, compounds **4a**–**4e** and **4i**–**4k**, 64–89%). Next, electron-rich substituted benzoic acids **3** (R = 4-OCH_3_, 4-CH_3_) were found to be compatible with *N*, *N*-dimethylanilines **2**, and N-isocyaniminotriphenylphosphorane **1**, affording the corresponding 1,3,4-oxadiazoles (**4f**–**4g**, **4m** and **4p**–**4q**, 65–88%). Notably, strongly electron-withdrawing substituted benzoic acid (R = 4-NO_2_) and aliphatic carboxylic acids (R^4^ = Et), as well as heterocyclic aromatic carboxylic acids, are particularly compatible with the optimization conditions to give the target products (**4l**, **4t** and **4s**, 64–80%). Different tertiary amines, such as *N*, *N*-diethylanilines, and *N*-methyl-*N*-phenylaniline, were also found to be suitable under the optimized conditions, affording the target compounds (**4h**, **4n**, **4r** and **4u**–**4w**, 64–83%). Fortunately, treating cyclic amines with high steric hindrance under the optimal conditions generated 1,3,4-oxadiazoles **4x** and **4y**, respectively, in 74–78% yields. 

**Scheme 3:**
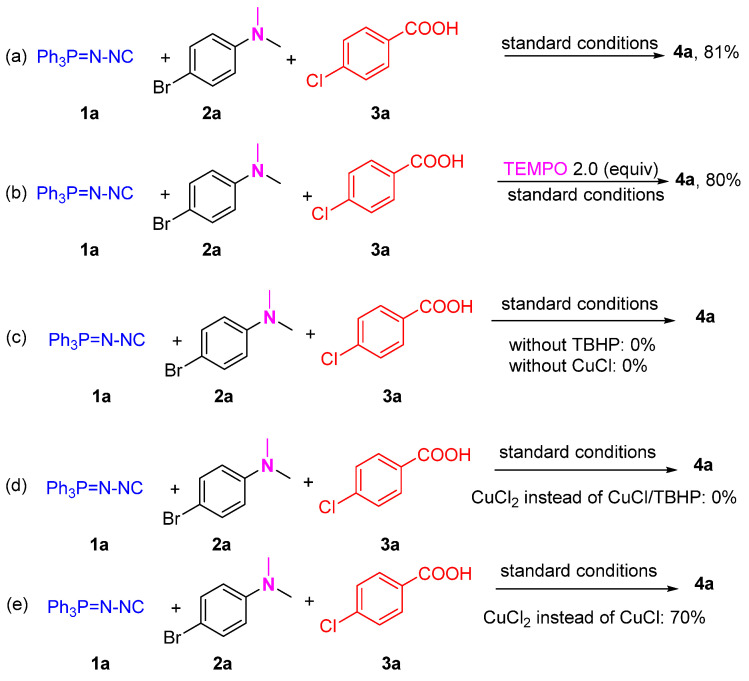
Control Experiments.

To investigate the mechanism of this tandem oxidative Ugi reaction, several control experiments were carried out (Scheme 3). Under typical conditions, the multicomponent reactions of **1**, **2a**, and **3a** produced **4a** with 81% yield (Scheme 3a). Following this, the reaction proceeded without any problems to produce the intended product **4a** in 80% of the cases where stoichiometric concentrations of radical quenchers 2,2,6,6-tetramethyl-1-piperidyloxy (TEMPO) were present (Scheme 3b). The results implied that this reaction may undergo a non-radical mechanism. Unsurprisingly, when this process was carried out only in the presence of peroxide TBHP or copper catalyst CuCl, it could be successfully completed (Scheme 3c). The above results showed that both the peroxide TBHP and the copper catalyst could play an important role in the reaction process. Next, we only used CuCl_2_ instead of CuCl/TBHP, and the final product **4a** could not be obtained (Scheme 3d). By replacing CuCl with CuCl_2_, the product yield was reduced to 70% (Scheme 3e). The experiments in Scheme 3d,e indicated that this process could be carried out with the catalysis of copper(II) and the presence of in situ-generated copper(II) catalysis can improve the rate of reaction. 

**Figure 2:**
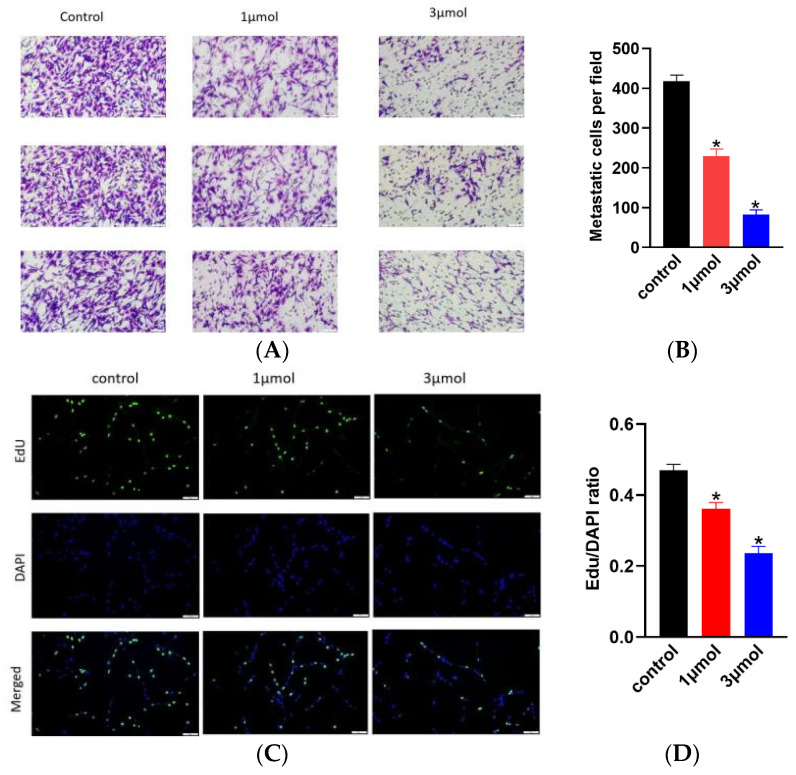
(**A**) Staining pictures of cell crystals in each group. (**B**) Statistical chart of the number of cloned cells in each group of plates (**C**) Fluorescence staining of EdU and DAPI. (**D**) Statistical chart of Edu positive cell rate. * *p* < 0.05 vs. control group.

To evaluate the potential of compound **4** as a lead drug, the anticancer activity of compound **4f** with the least cytotoxicity was tested. Transwell migration assays were conducted to evaluate the effect of compound **4f** on hepatoma cell migration; compared with the control group, the colony formation ability of cells in the 1 μM and 3 μM groups was significantly reduced (*p* < 0.05), indicating that compound **4f** treatment can significantly inhibit the migration of hepatoma cells in a dose-dependent manner (Figure 2A,B). Furthermore, the proliferation of hepatoma cells was determined by the EdU assay, and compound **4f** was applied to cells in different concentrations (0, 1, 3 μM) (Figure 2C,D). The results showed that an increase in the concentration of **4f** decreased the number of EdU-positive cells, which indicated that compound **4f** could effectively inhibit the proliferation of hepatoma cells.

**Scheme 4:**
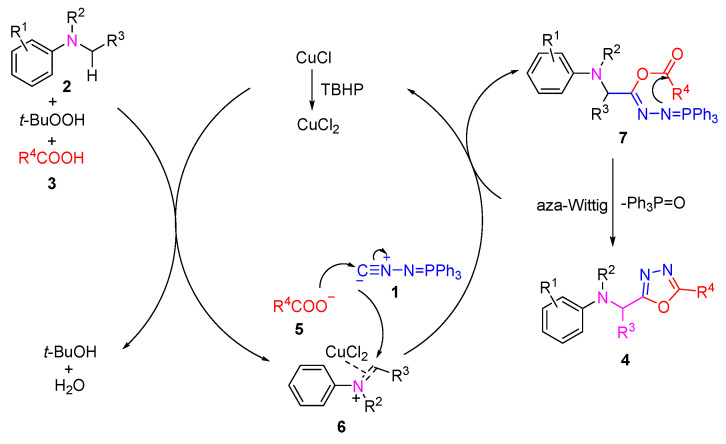
Proposed mechanism for formation of **4**.

Based on the aforementioned results and preceding literature [21,38], a plausible mechanism for this Cu(I)-catalyzed multicomponent process is delineated in Scheme 4. Initially, tertiary amine **2** was able to undergo a conversion to an iminium state through TBHP-mediated oxidation, followed by the in situ generation of CuCl_2_ via the oxidation of CuCl using TBHP. Subsequently, copper(II)–iminium complexes **6** were formed directly through the coordination catalysis of copper(II). These copper(II)–iminium complexes **6** then engaged in the Ugi-type reaction, leading to the formation of intermediate imidate **7**, while concomitantly liberating the copper species through successive attacks from isocyanide **1** and the conjugate base **5** of the acid. In parallel, the copper species persisted in participating in the subsequent catalytic cycle. Ultimately, imidate **7** underwent an intramolecular aza-Wittig reaction, culminating in the production of the desired product **4**.

## 3. Materials and Methods

### 3.1. General Information

Market-available methyl 2-formylbenzoate **2**, primary amines **3**, and carboxylic acids **4** were used directly without purification. As described in the literature [49], (N-isocyanimine)triphenylphosphorane **1** has been synthesized. All solvents and reagents were purchased from commercial sources, unless otherwise noted. Commercial reagents were used as supplied or purified by standard techniques wherever necessary. Column chromatography was performed using 200–300 mesh silica gel with the proper solvent system according to TLC analysis using I_2_ stain and UV light to visualize the reaction components. Melting points were determined using an X-4 model apparatus and were uncorrected. NMR were recorded in CDCl_3_ on a Bruker Avance III 400 spectrometer (Bruker Corporation, Billerica, MA, USA). Chemical shifts for ^l^H NMR spectra are reported in ppm using TMS as an internal reference (δ = 0). Chemical shifts for ^13^C NMR spectra were recorded in parts per million from TMS using the central peak of CDCl_3_ (77.0 ppm) as the internal standard. The terms m, s, d, t, q refer to multiplet, singlet, doublet, triplet, quartlet signals. Mass spectra were obtained on a SHIMADZU LCMS-8040 spectrometer (Shimadzu, Kyoto, Japan) with ESI. Elementary analyses were taken on a Vario EL III elementary analysis instrument.

### 3.2. General Procedure for the Synthesis of 1,3,4-Oxadiazoles ***4a***–***4y***

A fusion of CuCl (10 mg, 0.1 mmol) and *t*-butyl hydroperoxide (5~6 M TBHP in decane, 1.5 mmol) was meticulously introduced into a 25 mL three-neck flask that had been subjected to desiccation through the application of a heat gun under conditions of high-vacuum. Subsequently, the three-neck flask underwent evacuation and was then charged with a controlled influx of dry N_2_. The introduction of (N-isocyanimine) triphenylphosphorane 1 (302 mg, 1.0 mmol), tertiary amine 2 (2.0 mmol), carboxylic acid 3 (1.5 mmol), 4 Å MS (300 mg), and anhydrous CH_3_CN (5 mL) occurred in a sequential manner at the prevailing room temperature. The ensuing course of action involved elevating the reaction mixture to a temperature of 60 °C, with concurrent agitation maintained over a span of 8–12 h, during which the advancement of the reaction was meticulously tracked through the utilization of thin-layer chromatography (TLC). The ultimate step encompassed the removal of the solvent under conditions of reduced pressure, thereby leaving behind a residual substance. This residue was subsequently subjected to a purification process employing flash chromatography on silica gel, utilizing a mixture of petroleum ether and ethyl acetate in a volumetric ratio of 5:1 to 4:1, resulting in the acquisition of compound **4**.

4-bromo-N-((5-(4-chlorophenyl)-1,3,4-oxadiazol-2-yl)methyl)-N-methylaniline (4a): White solid (yield 0.307 g, 81%), mp 92–93 °C; ^1^H NMR (CDCl_3_, 400 MHz): δ (ppm) 7.92–7.89 (m, 2H), 7.48–7.32 (m, 4H), 6.78–6.74 (m, 2H), 4.73 (s, 2H), 3.11 (s, 3H); ^13^C{^1^H}NMR (CDCl_3_, 100 MHz): δ (ppm) 164.4, 163.5, 147.3, 138.1, 132.0, 129.4, 128.1, 122.0, 114.8, 110.4, 47.5, 39.1. LCMS (ESI) *m*/*z* [M+H]^+^: 378. Anal. Calcd for C_16_H_13_BrClN_3_O: C, 50.75; H, 3.46; N, 11.10; Found: C, 50.47; H, 3.16; N, 11.32.

4-bromo-N-((5-(4-bromophenyl)-1,3,4-oxadiazol-2-yl)methyl)-N-methylaniline (4b): White solid (yield 0.300 g, 71%), mp 92–93 °C; ^1^H NMR (CDCl_3_, 400 MHz): δ (ppm) 7.85–7.82 (m, 2H), 7.64–7.61 (m, 2H), 7.36–7.32 (m, 2H), 6.77–6.74 (m, 2H), 4.72 (s, 2H), 3.11 (s, 3H); ^13^C{^1^H}NMR (CDCl_3_, 100 MHz): δ (ppm) 164.5, 163.6, 147.3, 132.4, 132.0, 128.3, 126.6, 122.4, 114.8, 110.5, 47.6, 39.1. LCMS (ESI) *m*/*z* [M+H]^+^: 424. Anal. Calcd for C_16_H_13_Br_2_N_3_O: C, 45.42; H, 3.10; N, 9.93; Found: C, 45.18; H, 2.86; N, 9.75.

**4**-bromo-N-((5-(3-chlorophenyl)-1,3,4-oxadiazol-2-yl)methyl)-N-methylaniline (4c): White solid (yield 0.242 g, 64%), mp 86–87 °C; ^1^H NMR (CDCl_3_, 400 MHz): δ (ppm) 7.96–7.85 (m, 2H), 7.51–7.32 (m, 4H), 6.78–6.74 (m, 2H), 4.73 (s, 2H), 3.12 (s, 3H); ^13^C{^1^H}NMR (CDCl_3_, 100 MHz): δ (ppm) 164.1, 163.7, 147.3, 135.1, 132.0, 131.9, 130.4, 126.8, 125.1, 125.0, 114.8, 110.4, 47.6, 39.1. LCMS (ESI) *m*/*z* [M+H]^+^: 378. Anal. Calcd for C_16_H_13_BrClN_3_O: C, 50.75; H, 3.46; 9.36; N, 11.10; Found: C, 50.95; H, 3.26; N, 10.87.

4-bromo-N-((5-(2-bromophenyl)-1,3,4-oxadiazol-2-yl)methyl)-N-methylaniline (4d): White solid (yield 0.300 g, 71%), mp 87–88 °C; ^1^H NMR (CDCl_3_, 400 MHz): δ (ppm) 7.89–7.87 (m, 2H), 7.44–7.31 (m, 4H), 7.77–6.75 (m, 2H), 4.77 (s, 2H), 3.13 (s, 3H); ^13^C{^1^H}NMR (CDCl_3_, 100 MHz): δ (ppm) 164.2, 163.8, 147.2, 134.4, 132.6, 131.9, 131.6, 127.5, 124.9, 121.4, 114.8, 110.3, 47.5, 39.1. LCMS (ESI) *m*/*z* [M+H]^+^: 424. Anal. Calcd for C_16_H_13_Br_2_N_3_O: C, 45.42; H, 3.10; N, 9.93; Found: C, 45.14; H, 2.88; N, 9.70.

4-bromo-N-((5-(2-chlorophenyl)-1,3,4-oxadiazol-2-yl)methyl)-N-methylaniline (4e): White solid (yield 0.275 g, 73%), mp 85–86 °C; ^1^H NMR (CDCl_3_, 400 MHz): δ (ppm) 7.95–7.92 (m, 1H), 7.52–7.31 (m, 5H), 6.77–6.75 (m, 2H), 4.77 (s, 2H), 3.12 (s, 3H); ^13^C{^1^H}NMR (CDCl_3_, 100 MHz): δ (ppm) 163.8, 163.6, 147.2, 133.0, 132.5, 131.9, 131.1, 131.1, 127.0, 122.8, 114.8, 110.3, 47.5, 39.0. LCMS (ESI) *m*/*z* [M+H]^+^: 378. Anal. Calcd for C_16_H_13_BrClN_3_O: C, 50.75; H, 3.46; N, 11.10; Found: C, 50.55; H, 3.22; N, 10.95.

4-bromo-N-methyl-N-((5-(p-tolyl)-1,3,4-oxadiazol-2-yl)methyl)aniline (4f): White solid (yield 0.233 g, 65%), mp 97–98 °C; ^1^H NMR (CDCl_3_, 400 MHz): δ (ppm) 7.86 (d, *J* = 8.0 Hz, 2H), 7.35–7.27 (m, 4H), 6.77–6.75 (m, 2H), 4.71 (s, 2H), 3.11 (s, 3H), 2.41 (s, 3H); ^13^C{^1^H}NMR (CDCl_3_, 100 MHz): δ (ppm) 165.4, 163.1, 147.4, 142.4, 131.9, 129.7, 126.8, 120.8, 114.8, 110.3, 47.5, 39.0, 21.6. LCMS (ESI) *m*/*z* [M+H]^+^: 358. Anal. Calcd for C_17_H_16_BrN_3_O: C, 57.00; H, 4.50; N, 11.73; Found: C, 56.82; H, 4.62; N, 11.60.

4-bromo-N-((5-(4-methoxyphenyl)-1,3,4-oxadiazol-2-yl)methyl)-N-methylaniline (4g): White solid (yield 0.277 g, 74%), mp 108–109 °C; ^1^H NMR (CDCl_3_, 400 MHz): δ (ppm) 7.92–7.89 (m, 2H), 7.35–7.32 (m, 2H), 6.99–6.96 (m, 2H), 6.77–6.75 (m, 2H), 4.70 (s, 2H), 3.86 (s, 3H), 3.10 (s, 3H); ^13^C{^1^H}NMR (CDCl_3_, 100 MHz): δ (ppm) 165.1, 162.8, 162.3, 147.4, 131.9, 128.6, 116.0, 114.7, 114.4, 110.2, 55.4, 47.4, 38.9. LCMS (ESI) *m*/*z* [M+H]^+^: 374. Anal. Calcd for C_17_H_16_BrN_3_O_2_: C, 54.56; H, 4.31; N, 11.23; Found: C, 54.75; H, 4.11; N, 11.02.

4-bromo-N-methyl-N-((5-phenyl-1,3,4-oxadiazol-2-yl)methyl)aniline (4h): White solid (yield 0.220 g, 64%), mp 93–94 °C; ^1^H NMR (CDCl_3_, 400 MHz): δ (ppm) 7.99–7.96 (m, 2H), 7.52–7.32 (m, 5H), 6.78–6.74 (m, 2H), 4.73 (s, 2H), 3.11 (s, 3H); ^13^C{^1^H}NMR (CDCl_3_, 100 MHz): δ (ppm) 165.1, 163.3, 147.3, 131.9, 131.8, 128.9, 126.8, 123.5, 114.7, 110.2, 47.4, 39.0. LCMS (ESI) *m*/*z* [M+H]^+^: 344. Anal. Calcd for C_16_H_14_BrN_3_O: C, 55.83; H, 4.10; N, 12.21; Found: C, 55.57; H, 3.91; N, 12.01.

N-((5-(4-chlorophenyl)-1,3,4-oxadiazol-2-yl)methyl)-N,4-dimethylaniline (4i): White solid (yield 0.264 g, 84%), mp 88–89 °C; ^1^H NMR (CDCl_3_, 400 MHz): δ (ppm) 7.93–7.90 (m, 2H), 7.47–7.44 (m, 2H), 7.08 (d, *J* = 8.4 Hz, 2H), 6.84–6.81 (m, 2H), 4.71 (s, 2H), 3.09 (s, 3H), 2.26 (s, 3H); ^13^C{^1^H}NMR (CDCl_3_, 100 MHz): δ (ppm) 164.3, 164.1, 146.3, 137.9, 129.8, 129.3, 128.1, 127.7, 122.1, 113.6, 47.9, 39.1, 20.2. LCMS (ESI) *m*/*z* [M+H]^+^: 314. Anal. Calcd for C_17_H_16_ClN_3_O: C, 65.07; H, 5.14; N, 13.39; Found: C, 64.79; H, 4.94; N, 13.16.

N-((5-(4-bromophenyl)-1,3,4-oxadiazol-2-yl)methyl)-N,4-dimethylaniline (4j): White solid (yield 0.319 g, 89%), mp 93–94 °C; ^1^H NMR (CDCl_3_, 400 MHz): δ (ppm) 7.84 (d, *J* = 8.4 Hz, 2H), 7.61 (d, *J* = 8.4 Hz, 2H), 7.08 (d, *J* = 8.4 Hz, 2H), 6.82 (d, *J* = 8.4 Hz, 2H), 4.72 (s, 2H), 3.09 (s, 3H), 2.26 (s, 3H); ^13^C{^1^H}NMR (CDCl_3_, 100 MHz): δ (ppm) 164.4, 164.2, 146.3, 132.3, 129.8, 128.2, 127.7, 126.4, 122.6, 113.6, 48.0, 39.1, 20.2. LCMS (ESI) *m*/*z* [M+H]^+^: 358. Anal. Calcd for C_17_H_16_BrN_3_O: C, 57.00; H, 4.50; N, 11.73; Found: C, 56.80; H, 4.32; N, 11.59.

N-((5-(2-chlorophenyl)-1,3,4-oxadiazol-2-yl)methyl)-N,4-dimethylaniline (4k): White solid (yield 0.207 g, 66%), mp 77–78 °C; ^1^H NMR (CDCl_3_, 400 MHz): δ (ppm) 7.93–7.91 (m, 1H), 7.50–7.33 (m, 3H), 7.06 (d, *J* = 8.0 Hz, 2H), 6.83–6.81 (m, 2H), 4.75 (s, 2H), 3.10 (s, 3H), 2.25 (s, 3H); ^13^C{^1^H}NMR (CDCl_3_, 100 MHz): δ (ppm) 164.3, 163.5, 146.2, 133.0, 132.3, 131.1, 131.1, 129.7, 127.6, 127.0, 123.0, 113.6, 47.9, 39.1, 20.2. LCMS (ESI) *m*/*z* [M+H]^+^: 314. Anal. Calcd for C_17_H_16_ClN_3_O: C, 65.07; H, 5.14; N, 13.39; Found: C, 64.87; H, 4.90; N, 13.11.

N,4-dimethyl-N-((5-(4-nitrophenyl)-1,3,4-oxadiazol-2-yl)methyl)aniline (4l): White solid (yield 0.208 g, 64%), mp 110–112 °C; ^1^H NMR (CDCl_3_, 400 MHz): δ (ppm) 8.36–8.33 (m, 2H), 8.18–8.16 (m, 2H), 7.09 (d, *J* = 8.0 Hz, 2H), 6.83 (d, *J* = 8.4 Hz, 2H), 4.77 (s, 2H), 3.11 (s, 3H), 2.26 (s, 3H); ^13^C{^1^H}NMR (CDCl_3_, 100 MHz): δ (ppm) 165.1, 163.4, 149.5, 146.2, 129.9, 129.2, 128.0, 127.8, 124.3, 113.7, 48.1, 39.3, 20.3. LCMS (ESI) *m*/*z* [M+H]^+^: 325. Anal. Calcd for C_17_H_16_N_4_O_3_: C, 62.95; H, 4.97; N, 17.27; Found: C, 62.75; H, 4.71; N, 17.02.

N-((5-(4-methoxyphenyl)-1,3,4-oxadiazol-2-yl)methyl)-N,4-dimethylaniline (4m): White solid (yield 0.257 g, 83%), mp 82–83 °C; ^1^H NMR (CDCl_3_, 400 MHz): δ (ppm) 7.92–7.90 (m, 2H), 7.08–6.81 (m, 6H), 4.69 (s, 2H), 3.85 (s, 3H), 3.08 (s, 3H), 2.25 (s, 3H); ^13^C{^1^H}NMR (CDCl_3_, 100 MHz): δ (ppm) 165.0, 163.4, 162.2, 146.4, 129.7, 128.6, 127.5, 116.2, 114.3, 113.6, 55.4, 47.9, 39.0, 20.2. LCMS (ESI) *m*/*z* [M+H]^+^: 310. Anal. Calcd for C_18_H_19_N_3_O_2_: C, 69.88; H, 6.19; N, 13.58; Found: C, 69.66; H, 5.98; N, 13.29.

N,4-dimethyl-N-((5-phenyl-1,3,4-oxadiazol-2-yl)methyl)aniline (4n): White solid (yield 0.193 g, 69%), mp 77–78 °C; ^1^H NMR (CDCl_3_, 400 MHz): δ (ppm) 7.99–7.97 (m, 2H), 7.51–7.44 (m, 3H), 7.08 (d, *J* = 8.0 Hz, 2H), 6.84–6.82 (m, 2H), 4.72 (s, 2H), 3.09 (s, 3H), 2.26 (s, 3H); ^13^C{^1^H}NMR (CDCl_3_, 100 MHz): δ (ppm) 165.0, 163.9, 146.3, 131.6, 129.7, 128.9, 127.6, 126.8, 123.7, 113.6, 47.9, 39.1, 20.2. LCMS (ESI) *m*/*z* [M+H]^+^: 280. Anal. Calcd for C_17_H_17_N_3_O: C, 73.10; H, 6.13; N, 15.04; Found: C, 72.88; H, 5.90; N, 14.82.

N-((5-(2-chlorophenyl)-1,3,4-oxadiazol-2-yl)methyl)-N-methylaniline (4o): White solid (yield 0.201 g, 67%), mp 76–77 °C; ^1^H NMR (CDCl_3_, 400 MHz): δ (ppm) 7.93–7.91 (m, 1H), 7.50–7.24 (m, 5H), 6.91–6.78 (m, 3H), 4.79 (s, 2H), 3.14 (s, 3H); ^13^C{^1^H}NMR (CDCl_3_, 100 MHz): δ (ppm) 164.2, 163.5, 148.3, 133.0, 132.4, 131.1, 129.2, 127.0, 122.9, 118.2, 113.2, 47.5, 38.9. LCMS (ESI) *m*/*z* [M+H]^+^: 300. Anal. Calcd for C_16_H_14_ClN_3_O: C, 64.11; H, 4.71; N, 14.02; Found: C, 63.90; H, 4.51; N, 13.78.

N-methyl-N-((5-(p-tolyl)-1,3,4-oxadiazol-2-yl)methyl)aniline (4p): White solid (yield 0.215 g, 77%), mp 101–102 °C; ^1^H NMR (CDCl_3_, 400 MHz): δ (ppm) 7.87–7.85 (m, 2H), 7.29–7.25 (m, 4H), 6.92–6.79 (m, 3H), 4.74 (s, 2H), 3.13 (s, 3H), 2.40 (s, 3H); ^13^C{^1^H}NMR (CDCl_3_, 100 MHz): δ (ppm) 165.3, 163.6, 148.5, 142.3, 129.6, 129.3, 126.8, 120.9, 118.2, 113.2, 47.6, 38.9, 21.6. LCMS (ESI) *m*/*z* [M+H]^+^: 280. Anal. Calcd for C_17_H_17_N_3_O: C, 73.10; H, 6.13; N, 15.04; Found: C, 72.94; H, 5.90; N, 14.89.

N-((5-(4-methoxyphenyl)-1,3,4-oxadiazol-2-yl)methyl)-N-methylaniline (4q): White solid (yield 0.260 g, 88%), mp 94–95 °C; ^1^H NMR (CDCl_3_, 400 MHz): δ (ppm) 7.92–7.89 (m, 2H), 7.28–7.24 (m, 2H), 6.97–6.78 (m, 5H), 4.72 (s, 2H), 3.84 (s, 3H), 3.11 (s, 3H); ^13^C{^1^H}NMR (CDCl_3_, 100 MHz): δ (ppm) 165.0, 163.3, 162.2, 148.4, 129.2, 128.6, 118.1, 116.1, 114.3, 113.2, 55.3, 47.6, 38.8. LCMS (ESI) *m*/*z* [M+H]^+^: 296. Anal. Calcd for C_17_H_17_N_3_O_2_: C, 69.14; H, 5.80; N, 14.23; Found: C, 68.91; H, 5.61; N, 14.03.

N-methyl-N-((5-phenyl-1,3,4-oxadiazol-2-yl)methyl)aniline (4r): White solid (yield 0.183 g, 69%), mp 97–98 °C; ^1^H NMR (CDCl_3_, 400 MHz): δ (ppm) 8.00–7.96 (m, 2H), 7.51–7.25 (m, 5H), 6.92–6.79 (m, 3H), 4.76 (s, 2H), 3.13 (s, 3H); ^13^C{^1^H}NMR (CDCl_3_, 100 MHz): δ (ppm) 165.2, 163.9, 148.4, 131.7, 129.3, 129.0, 126.9, 123.7, 118.3, 113.2, 47.7, 38.9. LCMS (ESI) *m*/*z* [M+H]^+^: 266. Anal. Calcd for C_16_H_15_N_3_O: C, 72.43; H, 5.70; N, 15.84; Found: C, 72.23; H, 5.49; N, 15.97.

N-methyl-N-((5-(thiophen-2-yl)-1,3,4-oxadiazol-2-yl)methyl)aniline (4s): White solid (yield 0.198 g, 73%), mp 94–95 °C; ^1^H NMR (CDCl_3_, 400 MHz): δ (ppm) 7.68–7.51 (m, 2H), 7.29–7.12 (m, 3H), 6.91–6.79 (m, 3H), 4.74 (s, 2H), 3.12 (s, 3H); ^13^C{^1^H}NMR (CDCl_3_, 100 MHz): δ (ppm) 163.2, 161.4, 148.3, 130.2, 129.9, 129.3, 128.0, 124.9, 118.3, 113.2, 47.6, 38.9. LCMS (ESI) *m*/*z* [M+H]^+^: 272. Anal. Calcd for C_14_H_13_N_3_OS: C, 61.97; H, 4.83; N, 15.49; Found: C, 61.77; H, 4.53; N, 15.22.

N-((5-ethyl-1,3,4-oxadiazol-2-yl)methyl)-N-methylaniline (4t): Colorless oil (yield 0.174 g, 80%), ^1^H NMR (CDCl_3_, 400 MHz): δ (ppm) 7.27–7.23 (m, 2H), 6.86–6.77 (m, 3H), 4.64 (s, 2H), 3.07 (s, 3H), 2.81 (q, *J* = 7.6 Hz, 2H), 1.32 (t, *J* = 7.6 Hz, 3H); ^13^C{^1^H}NMR (CDCl_3_, 100 MHz): δ (ppm) 168.2, 163.8, 148.4, 129.1, 118.0, 113.0, 47.5, 38.7, 18.9, 10.5. LCMS (ESI) *m*/*z* [M+H]^+^: 218 Anal. Calcd for C_13_H_17_N_3_O: C, 66.34; H, 6.96; N, 19.34; Found: C, 66.59; H, 6.76; N, 19.42.

N-((5-(4-methoxyphenyl)-1,3,4-oxadiazol-2-yl)methyl)-N,3-dimethylaniline (4u): White solid (yield 0.223 g, 72%), mp 90–91 °C; ^1^H NMR (CDCl_3_, 400 MHz): δ (ppm) 7.92–7.90 (m, 2H), 7.17–7.13 (m, 1H), 6.97–6.62 (m, 5H), 4.72 (s, 2H), 3.85 (s, 3H), 3.11 (s, 3H), 2.32 (s, 3H); ^13^C{^1^H}NMR (CDCl_3_, 100 MHz): δ (ppm) 165.0, 163.4, 162.2, 148.5, 138.9, 129.0, 128.6, 119.1, 116.1, 114.3, 114.0, 110.4, 55.3, 47.6, 38.8, 21.8. LCMS (ESI) *m*/*z* [M+H]^+^: 310. Anal. Calcd for C_18_H_19_N_3_O_2_: C, 69.88; H, 6.19; N, 13.58; Found: C, 69.25; H, 5.92; N, 13.28.

2-chloro-N-((5-(4-methoxyphenyl)-1,3,4-oxadiazol-2-yl)methyl)-N-methylaniline (4v): Colorless oil (yield 0.234 g, 71%), ^1^H NMR (CDCl_3_, 400 MHz): δ (ppm) 7.90–7.87 (m, 2H), 7.42–6.96 (m, 6H), 4.58 (s, 2H), 3.85 (s, 3H), 2.92 (s, 3H); ^13^C{^1^H}NMR (CDCl_3_, 100 MHz): δ (ppm) 165.1, 162.9, 162.2, 147.5, 130.7, 128.5, 128.5, 127.4, 124.4, 122.0, 116.1, 114.3, 55.3, 49.6, 40.3. LCMS (ESI) *m*/*z* [M+H]^+^: 330. Anal. Calcd for C_17_H_16_ClN_3_O_2_: C, 61.92; H, 4.89; N, 12.74; Found: C, 61.71; H, 4.65; N, 12.54.

N-((5-(4-chlorophenyl)-1,3,4-oxadiazol-2-yl)methyl)-N-phenylaniline (4w): White solid (yield 0.300 g, 83%), mp 85–86 °C; ^1^H NMR (CDCl_3_, 400 MHz): δ (ppm) 7.90–7.87 (m, 2H), 7.46–7.01 (m, 12H), 5.17 (s, 2H); ^13^C{^1^H}NMR (CDCl_3_, 100 MHz): δ (ppm) 164.3, 164.2, 147.0, 138.0, 129.5, 129.4, 128.1, 122.5, 122.1, 121.0, 47.3. LCMS (ESI) *m*/*z* [M+H]^+^: 362. Anal. Calcd for C_21_H_16_ClN_3_O: C, 69.71; H, 4.46; N, 11.61; Found: C, 69.52; H, 4.23; N, 11.37.

2-(4-methoxyphenyl)-5-(1-phenylpiperidin-2-yl)-1,3,4-oxadiazole (4x): White solid (yield 0.262 g, 78%), mp 110–111 °C; ^1^H NMR (CDCl_3_, 400 MHz): δ (ppm) 7.81 (d, *J* = 8.0 Hz, 2H), 7.26–6.83 (m, 7H), 5.24 (t, *J* = 4.4 Hz, 1H), 3.83 (s, 3H), 3.51–3.25 (m, 2H), 2.33–1.75 (m, 6H); ^13^C{^1^H}NMR (CDCl_3_, 100 MHz): δ (ppm) 165.9, 164.3, 162.1, 150.5, 129.1, 128.5, 120.1, 117.0, 116.3, 114.3, 55.3, 53.3, 45.7, 28.9, 25.2, 20.0. LCMS (ESI) *m*/*z* [M+H]^+^: 336. Anal. Calcd for C_20_H_21_N_3_O_2_: C, 71.62; H, 6.31; N, 12.53; Found: C, 71.40; H, 6.10; N, 12.43.

2-phenyl-5-(1-phenylpiperidin-2-yl)-1,3,4-oxadiazole (4y): White solid (yield 0.226 g, 74%), mp 121–122 °C; ^1^H NMR (CDCl_3_, 400 MHz): δ (ppm) 7.89–7.87 (m, 2H), 7.78–7.41 (m, 3H), 7.27–7.23 (m, 2H), 7.01 (d, *J* = 8.4 Hz, 2H), 6.85 (t, *J* = 7.2 Hz, 1H), 5.27 (t, *J* = 4.4 Hz, 1H), 4.52–3.27 (m, 2H), 2.35–1.77 (m, 6H); ^13^C{^1^H}NMR (CDCl_3_, 100 MHz): δ (ppm) 166.4, 164.4, 150.4, 131.5, 129.1, 128.9, 126.7, 123.8, 120.2, 117.1, 53.3, 45.7, 28.9, 25.2, 20.0. LCMS (ESI) *m*/*z* [M+H]^+^: 306. Anal. Calcd for C_19_H_19_N_3_O: C, 74.73; H, 6.27; N, 13.76; Found: C, 74.93; H, 6.00; N, 13.52.

## 4. Conclusions

In conclusion, we have documented a novel one-step Cu(I)/TBHP-mediated oxidative Ugi/aza-Wittig reaction for the synthesis of 1,3,4-oxadiazoles, derived from (N-isocyanimine)triphenylphosphorane, tertiary amines, and carboxylic acids, entailing the direct functionalization of amines’ α-carbon atoms. Remarkably, this process represents the inaugural instance of disubstituted 1,3,4-oxadiazoles synthesized from tertiary amines through the use of copper (I) catalysis and TBHP. Under mild conditions, this synthesis method demonstrated a high tolerance towards functional groups and a wide range of substrates. In addition, the biological evaluation indicates that compound **4f** is a promising starting point for the development of a drug for hepatoma, which makes it attractive for application in synthetic and medicinal chemistry.

## Figures and Tables

**Table 1:** Optimization of the formation of 1,3,4-oxadiazoles **4a**.

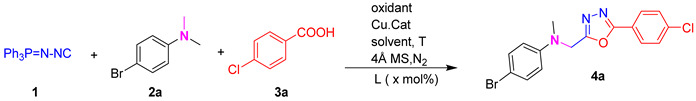						
Entry	Catalyst (mol%)	L (mol%)	Solvent	Oxidant	Temp (°C)	Yield of 4a (%) ^a^
1	CuCl (10)	2,2′-bipyridyl (10)	CH_3_CN	TBHP	60	79
2	CuBr_2_ (10)	2,2′-bipyridyl (10)	CH_3_CN	TBHP	60	65
3	CuI (10)	2,2′-bipyridyl (10)	CH_3_CN	TBHP	60	72
4	CuBr (10)	2,2′-bipyridyl (10)	CH_3_CN	TBHP	60	68
**5**	CuCl (10)	-	CH_3_CN	TBHP	60	81
6 ^b^	CuCl (10)	-	CH_3_CN	TBHP	60	66
7	CuCl (10)	-	PhMe	TBHP	60	38
8	CuCl (10)	-	MeOH	TBHP	60	24
9	CuCl (10)	-	THF	TBHP	60	32
10	CuCl (10)	-	CH_2_Cl_2_	TBHP	60	69
11	CuCl (10)	-	1,4-dioxane	TBHP	60	45
12 ^c^	CuCl (10)	-	CH_3_CN	BOP	60	65
13 ^d^	CuCl (10)	-	CH_3_CN	DTBP	60	52
14 ^e^	CuCl (10)	-	CH_3_CN	TBPB	60	38
15	CuCl (10)	-	CH_3_CN	TBHP	25	58
16	CuCl (10)	-	CH_3_CN	TBHP	40	69
17	CuCl (10)	-	CH_3_CN	TBHP	80	67
18	CuCl (5)	-	CH_3_CN	TBHP	60	69
19 ^f^	CuCl (10)	-	CH_3_CN	TBHP	60	80
20 ^g^	CuCl (10)	-	CH_3_CN	TBHP	60	77

^a^ *N*-isocyaniminotriphenylphosphorane **1** (1.0 mmol), 4-bromo-*N*, *N*-dimethylaniline **2a** (2.0 mmol), 4-Chlorobenzoicacid **3a** (1.5 mmol), *t*-butyl hydroperoxide (TBHP in decane, 1.5 mmol), copper catalysts (0.1 mmol), solvent (5.0 mL), 4Å MS (300 mg), N_2_, 60 °C, 12 h; ^b^ Air ambience; ^c^ BOP (1.5 mmol) = Benzoyl peroxide; ^d^ DTBP (1.5 mmol) = Di-tert-butyl peroxide; ^e^ TBPB (1.5 mmol) = tert-Butyl peroxybenzoate. ^f^ Reaction time: 24 h; ^g^ Microwave heating, 12 h.

## Data Availability

Data are contained within the article and Supplementary Materials.

## Supplementary Materials

The following supporting information can be downloaded at: https://www.mdpi.com/article/10.3390/molecules29061253/s1, ^1^H-NMR and ^13^C-NMR of compounds 4a–y.
