# Supplementary material for: One-Step Construction of 1,3,4-Oxadiazoles with Anticancer Activity from Tertiary Amines via a Sequential Copper(I)-Catalyzed Oxidative Ugi/aza-Wittig Reaction
Source: Molecules. 2024 Mar 12;29(6):1253. doi: 10.3390/molecules29061253 (PMC10975333; doi:10.3390/molecules29061253)

Supporting Information

One-Step Construction of 1,3,4-Oxadiazoles with Anticancer Activity from Tertiary Amines via a Sequential Copper(I)-Catalyzed Oxidative Ugi/aza-Wittig Reaction

Mei Sun [1,†], Nong-Qi Mao [2,†], Sheng-Long Wang [2,†], Xin-Ming Han [2], Gang Yao [2], Ping Xue [2], Chong-Yang Zeng [1], Yu-Ting Liu [1], Kai Chen [1], Xiao-Qing Gao [1] and Jun Xiong [2,*]

[1] School of Chemistry and Material Engineering, Anhui Key Laboratory of Low Temperature Co-Fired Materials, Huainan Normal University, Huainan 232038, China

[2] School of Pharmacy, Xianning Medical College, Hubei University of Science and Technology, Xianning 437100, China

* Correspondence: junxiong@hbust.edu.cn

† These authors contributed equally to this work.

Table of Contents

1. Copies of ^{1}H and ^{13}C NMR spectrum of compound **4a-4y** ……………………..…..S2-S26

4. Copies of ^{1}H and ^{13}C NMR spectrum of compound 4a-4y

^{1}H NMR (400 MHz, CDCl$_3$) of compound **4a**

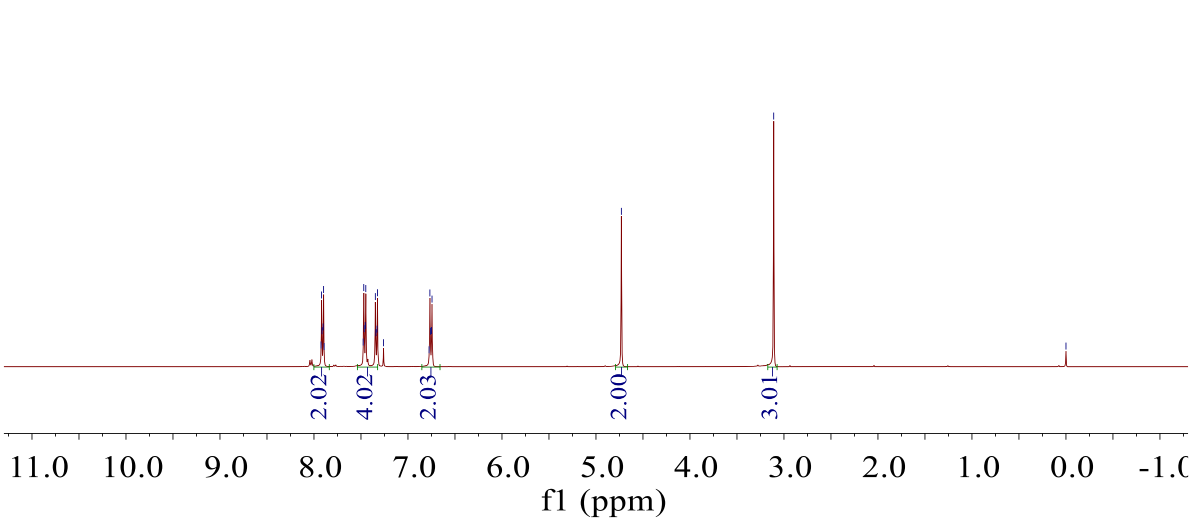

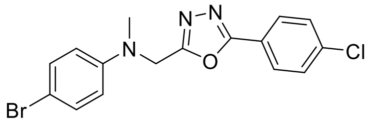

4a

^{13}C{^{1}H} NMR (100 MHz, CDCl$_3$) of compound **4a**

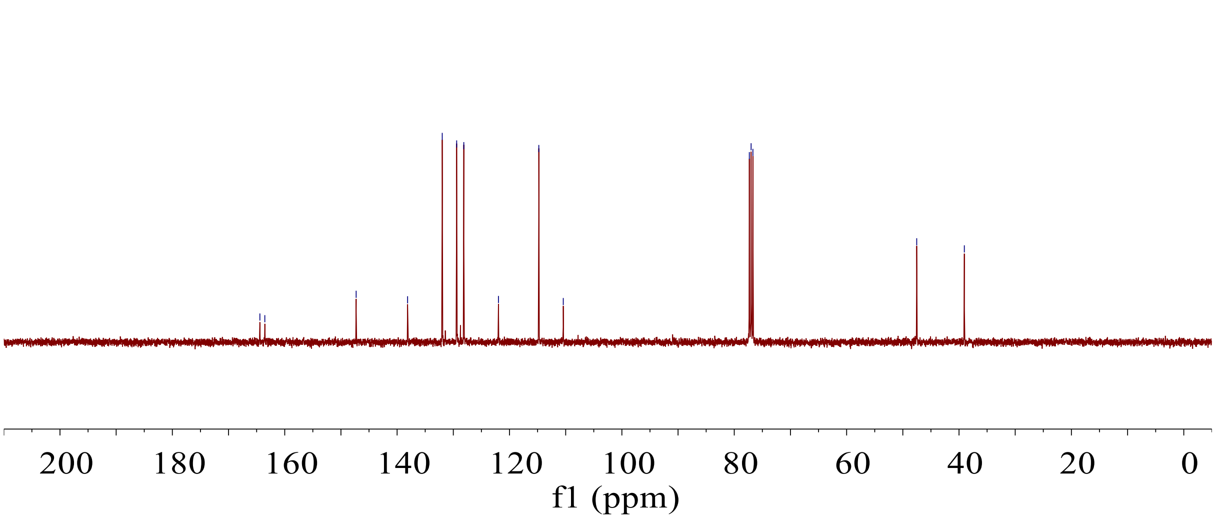

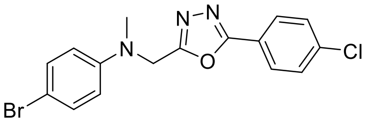

4a

^{1}H NMR (400 MHz, $CDCl_3$) of compound **4b**

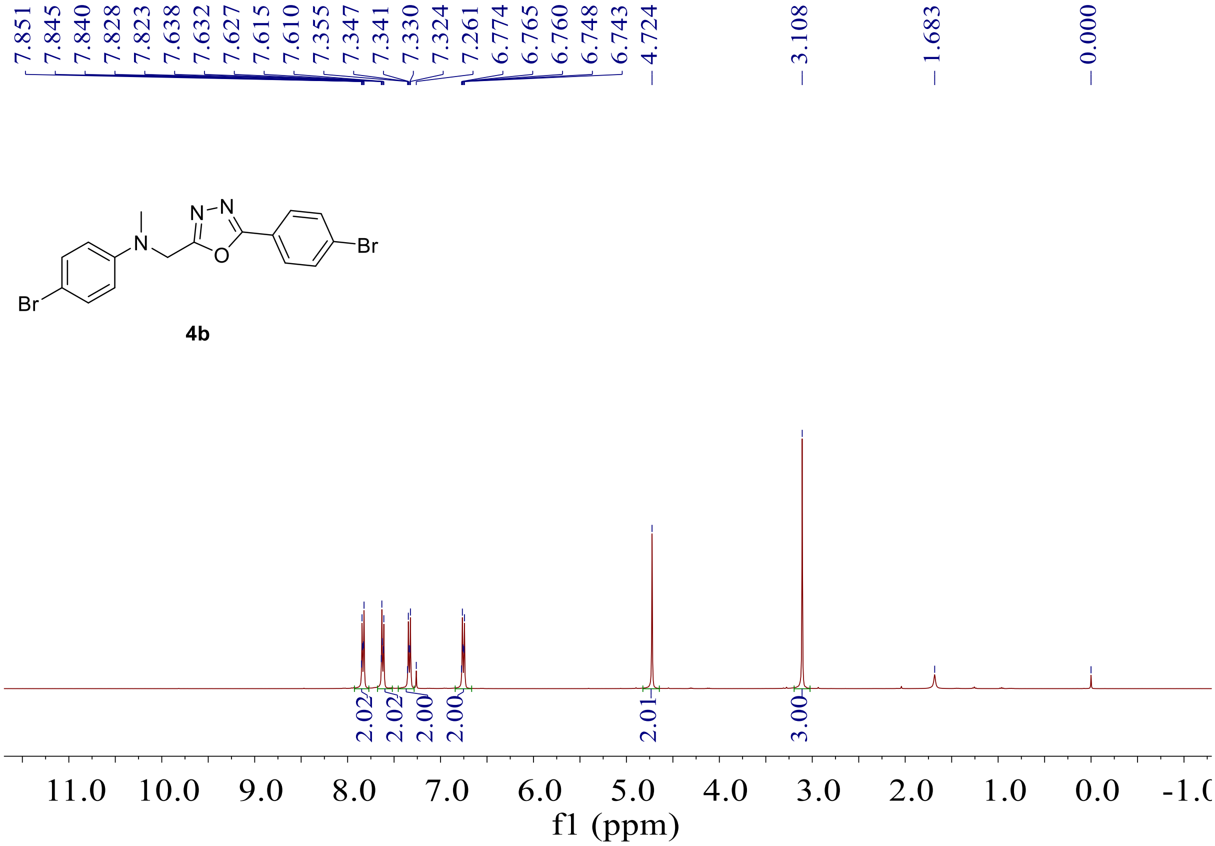

^{13}C{^{1}H} NMR (100 MHz, $CDCl_3$) of compound **4b**

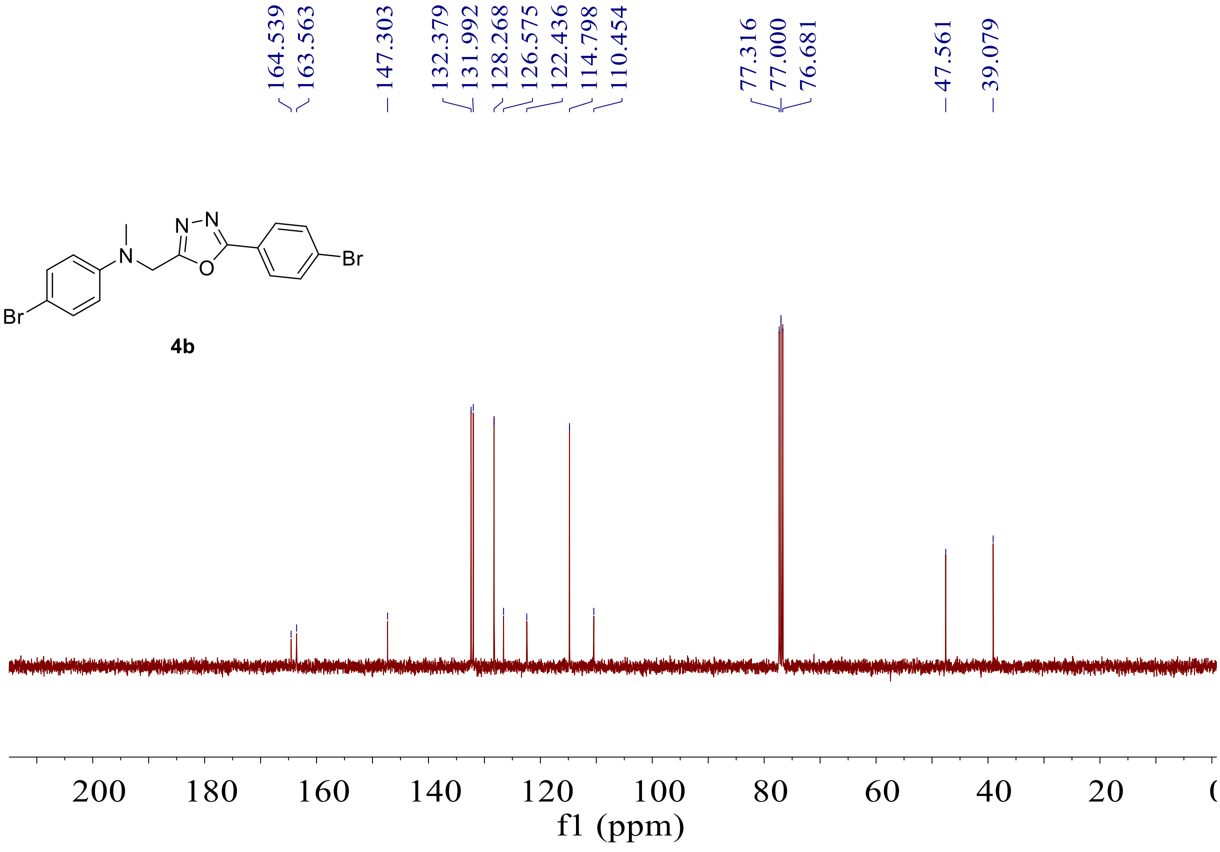

^{1}H NMR (400 MHz, $CDCl_3$) of compound **4c**

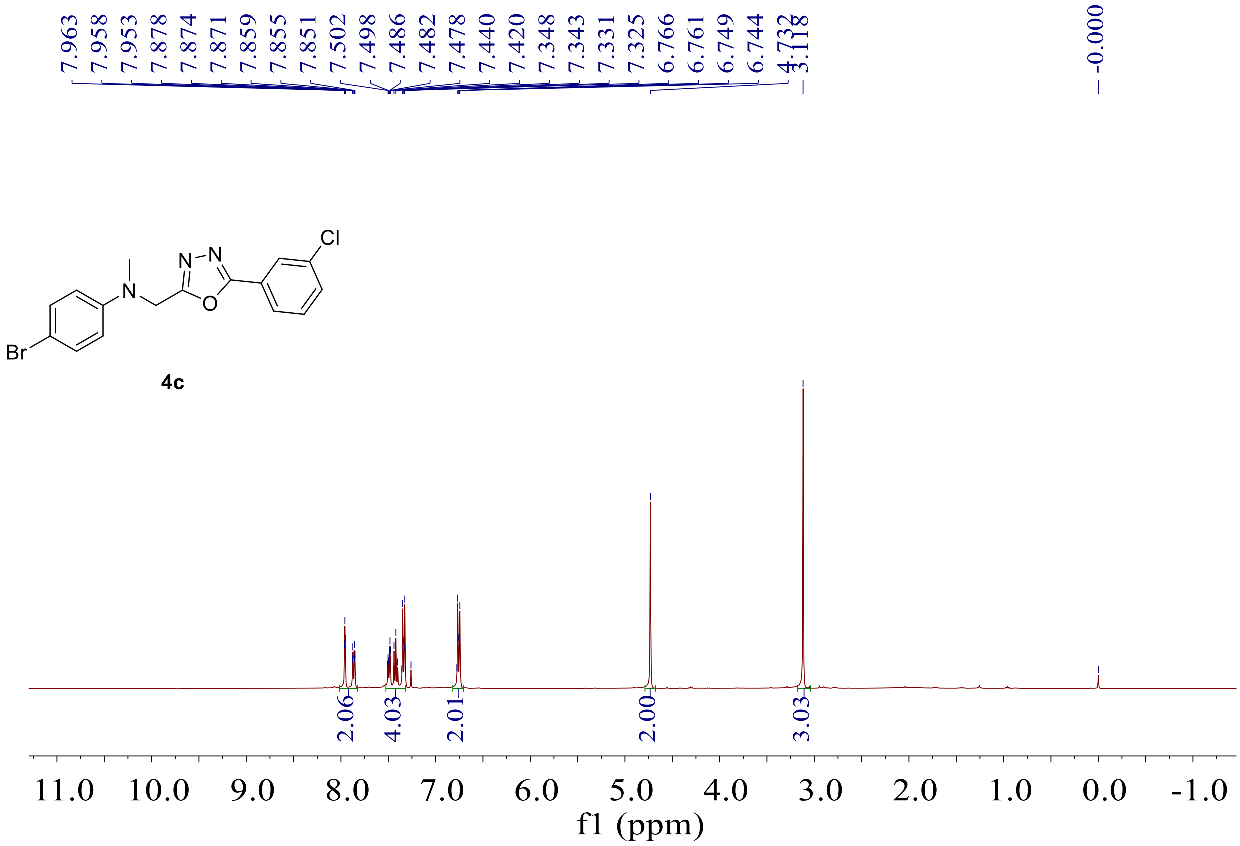

$^{13}C\{^{1}H\}$ NMR (100 MHz, $CDCl_3$) of compound **4c**

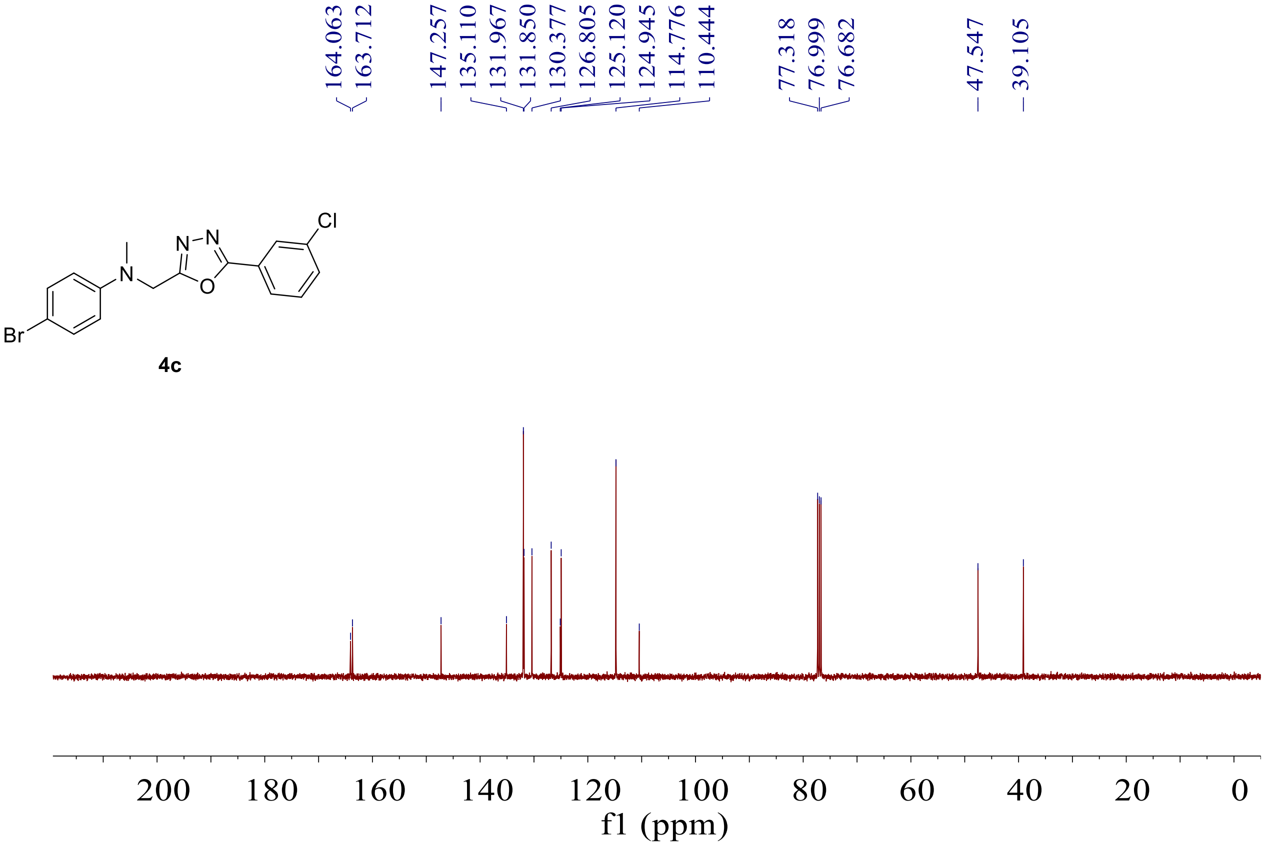

^{1}H NMR (400 MHz, $CDCl_3$) of compound **4d**

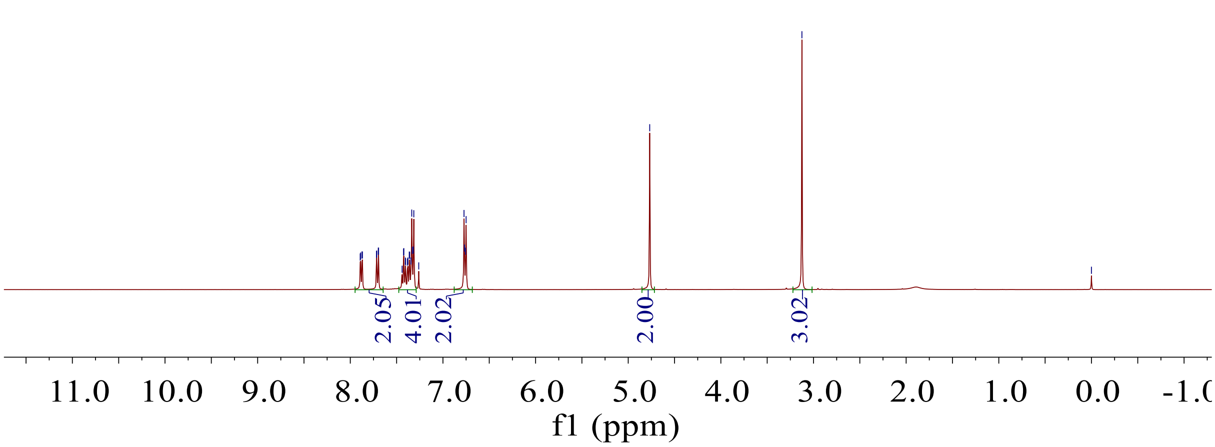

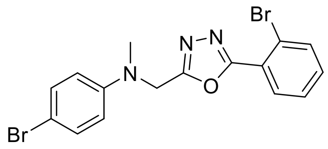

4d

$^{13}C\{^{1}H\}$ NMR (100 MHz, $CDCl_3$) of compound **4d**

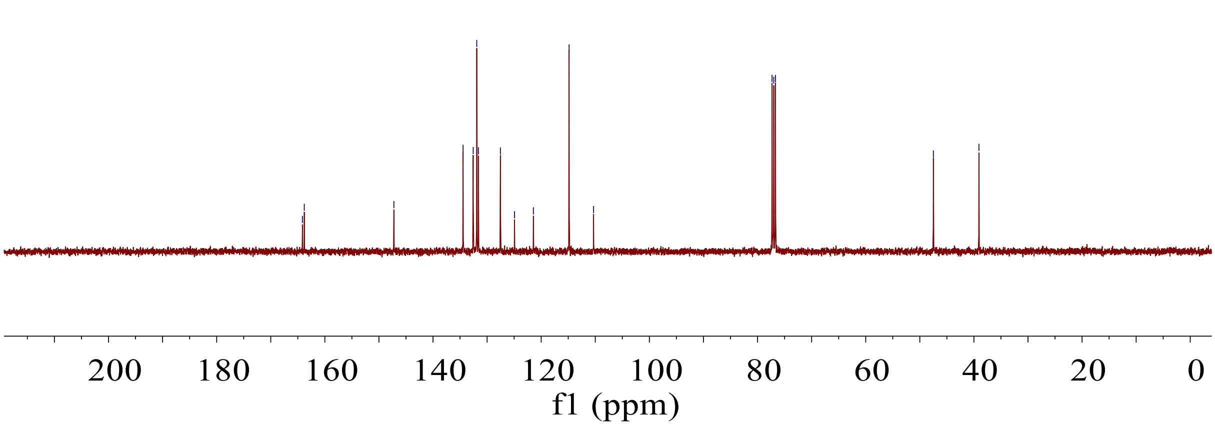

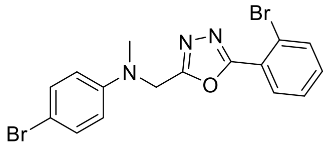

4d

^{1}H NMR (400 MHz, $CDCl_3$) of compound **4e**

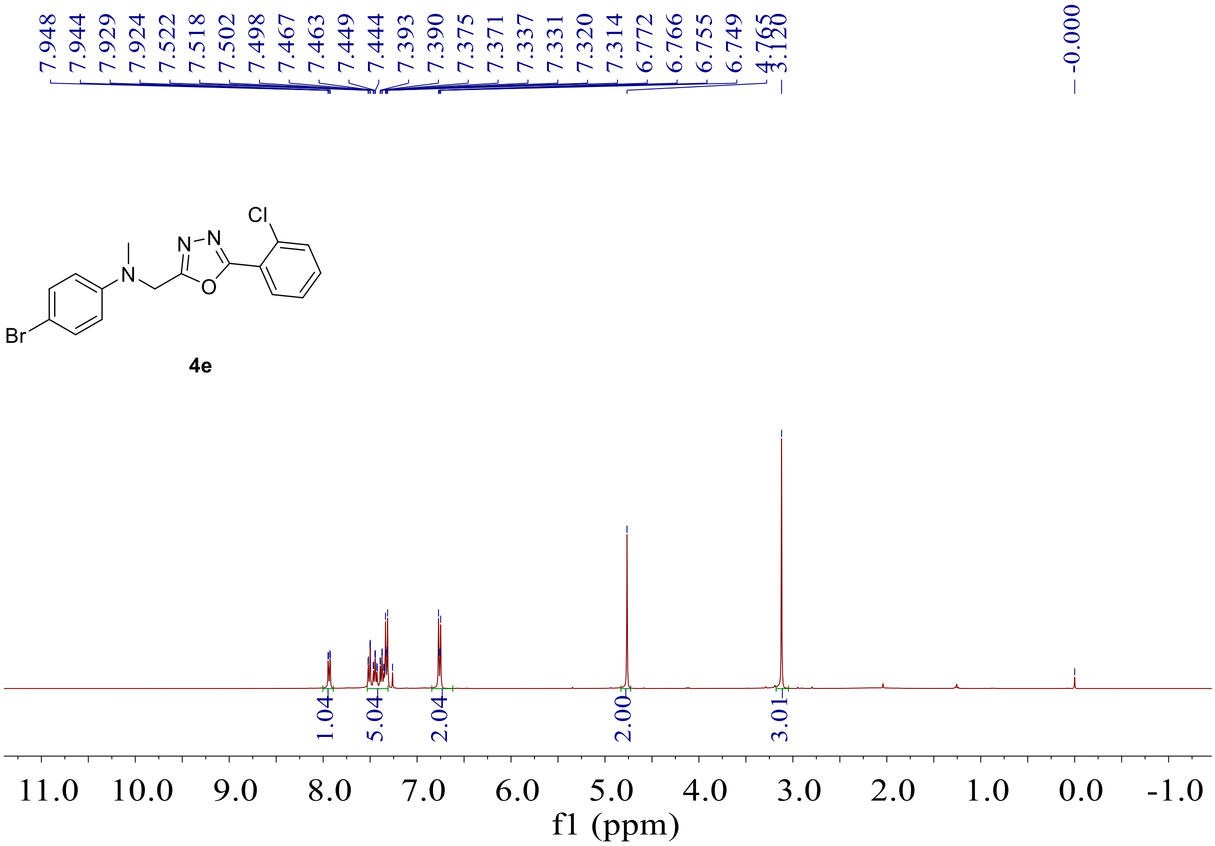

^{13}C{^{1}H} NMR (100 MHz, $CDCl_3$) of compound **4e**

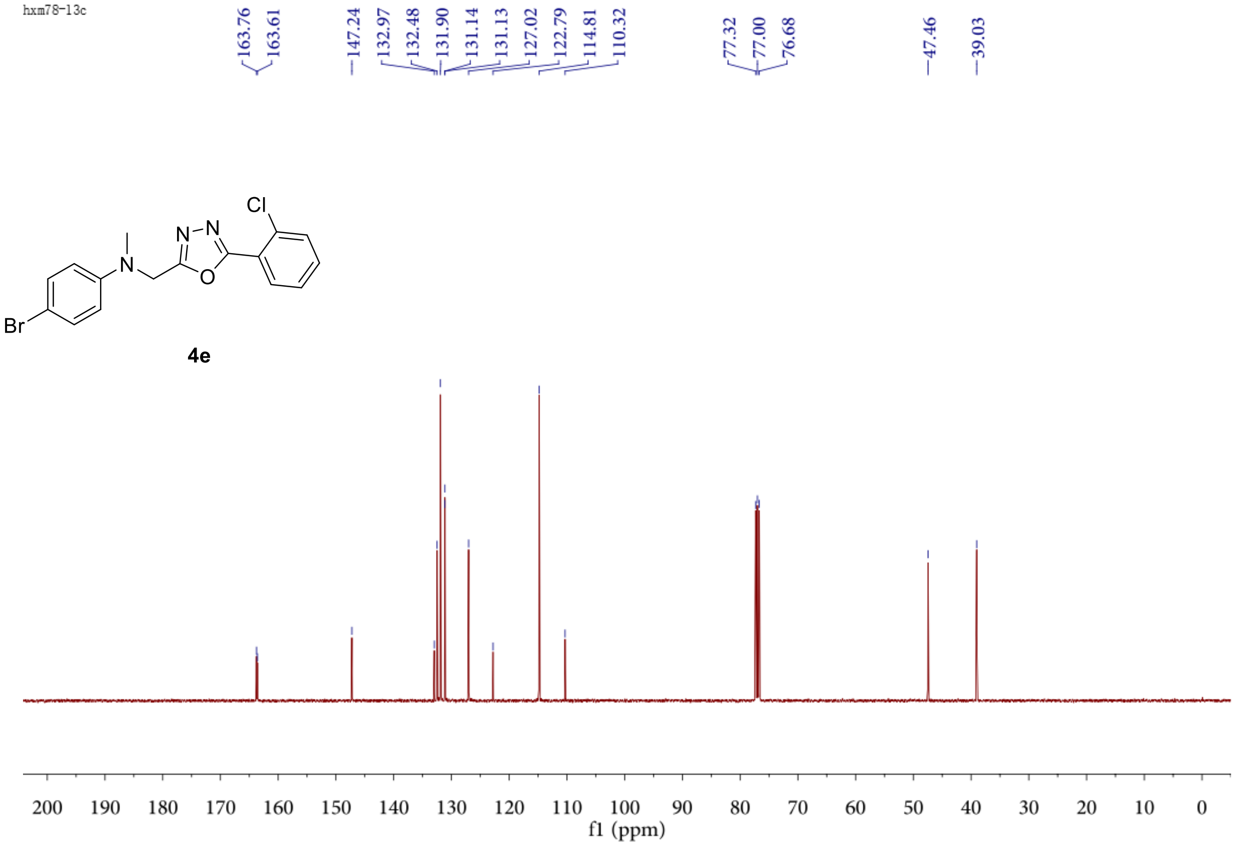

^{1}H NMR (400 MHz, $CDCl_3$) of compound **4f**

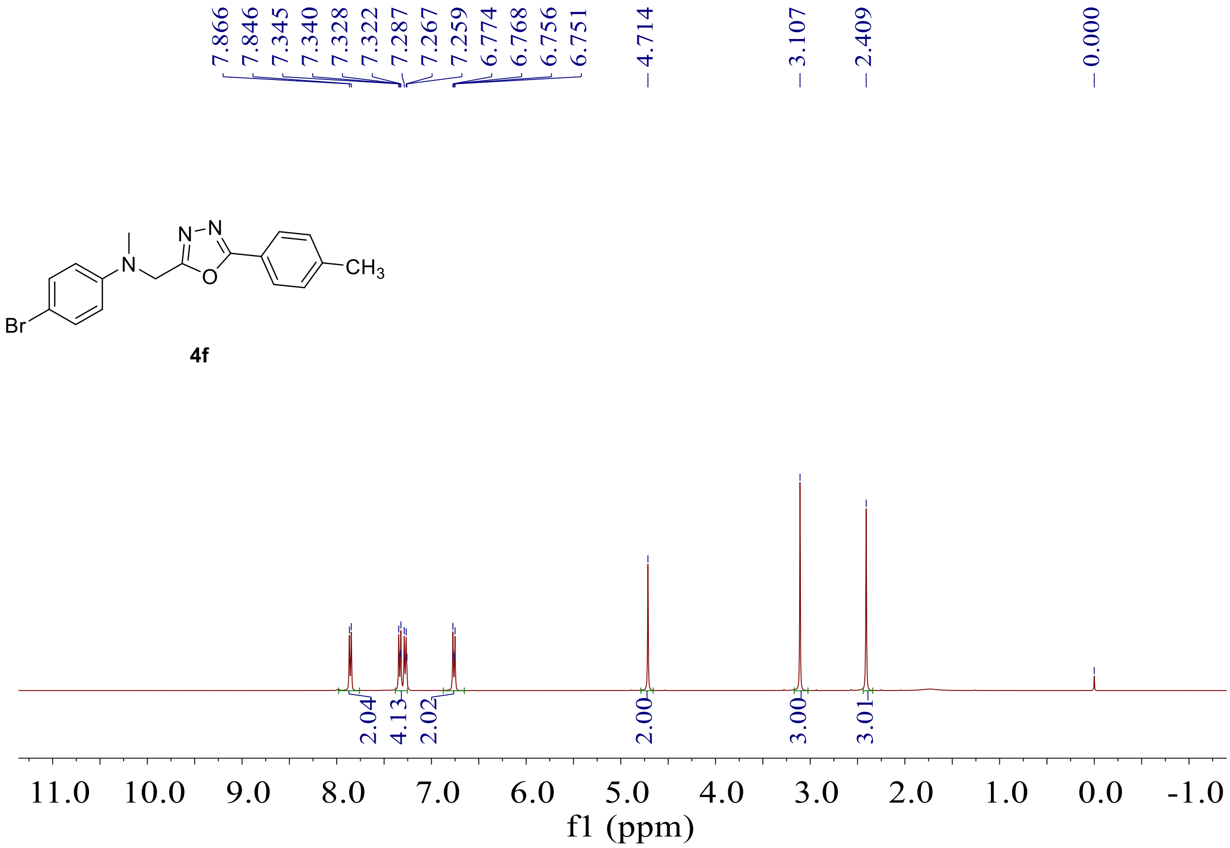

^{13}C{^{1}H} NMR (100 MHz, $CDCl_3$) of compound **4f**

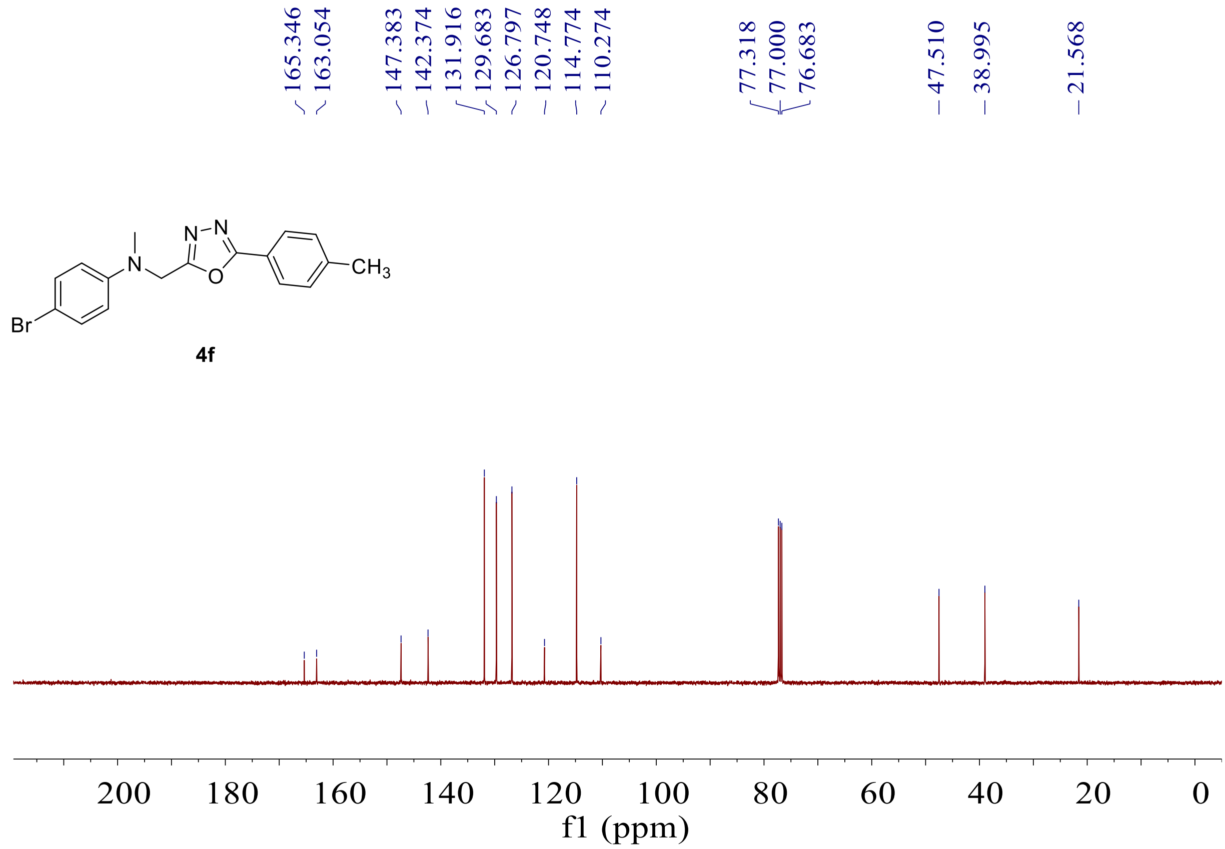

^{1}H NMR (400 MHz, $CDCl_3$) of compound **4g**

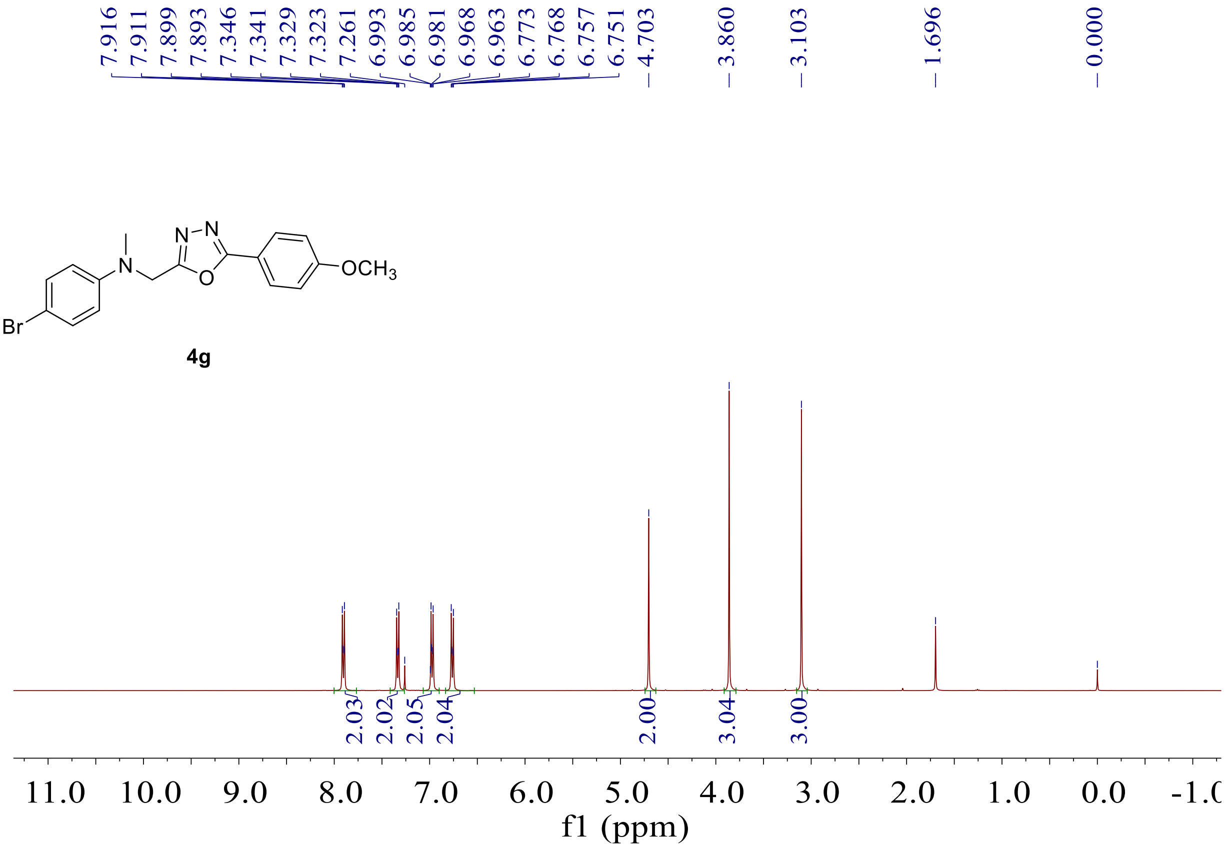

$^{13}C\{^{1}H\}$ NMR (100 MHz, $CDCl_3$) of compound **4g**

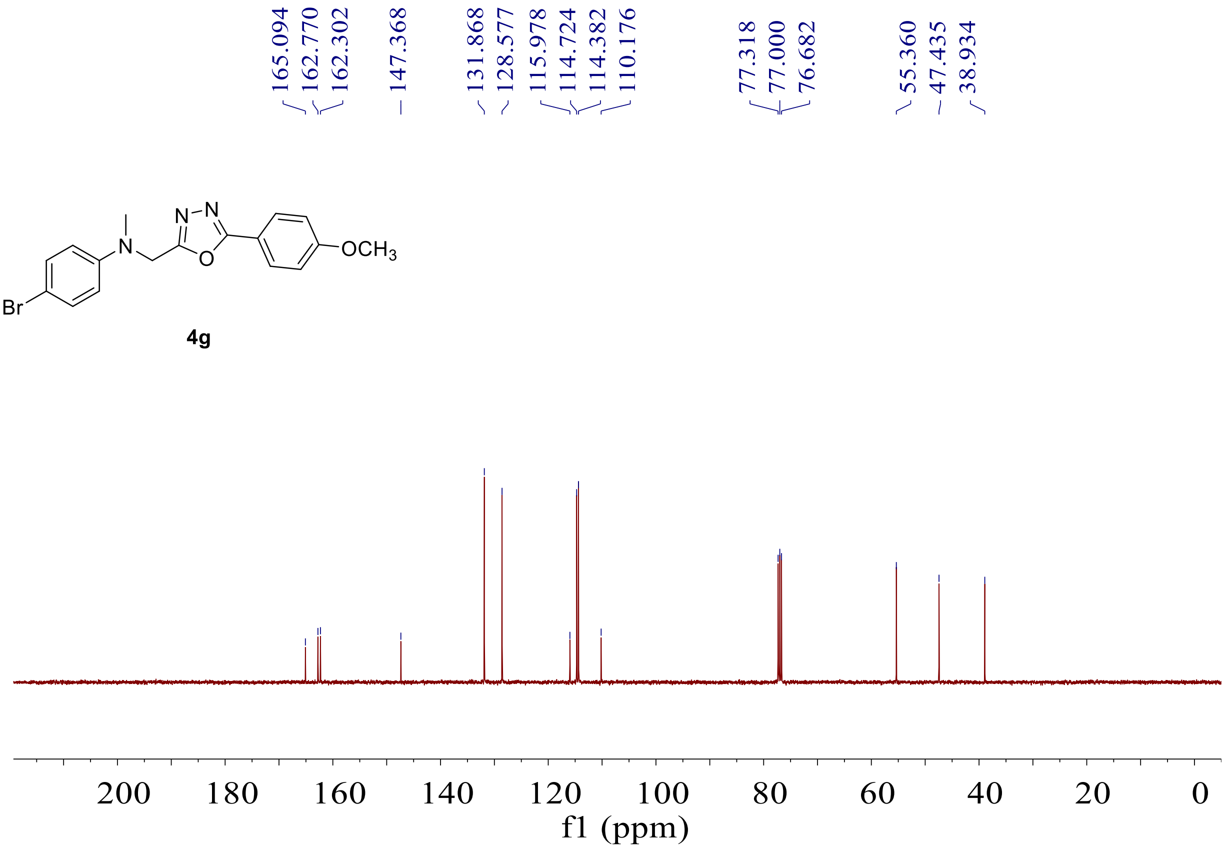

^{1}H NMR (400 MHz, $CDCl_3$) of compound **4h**

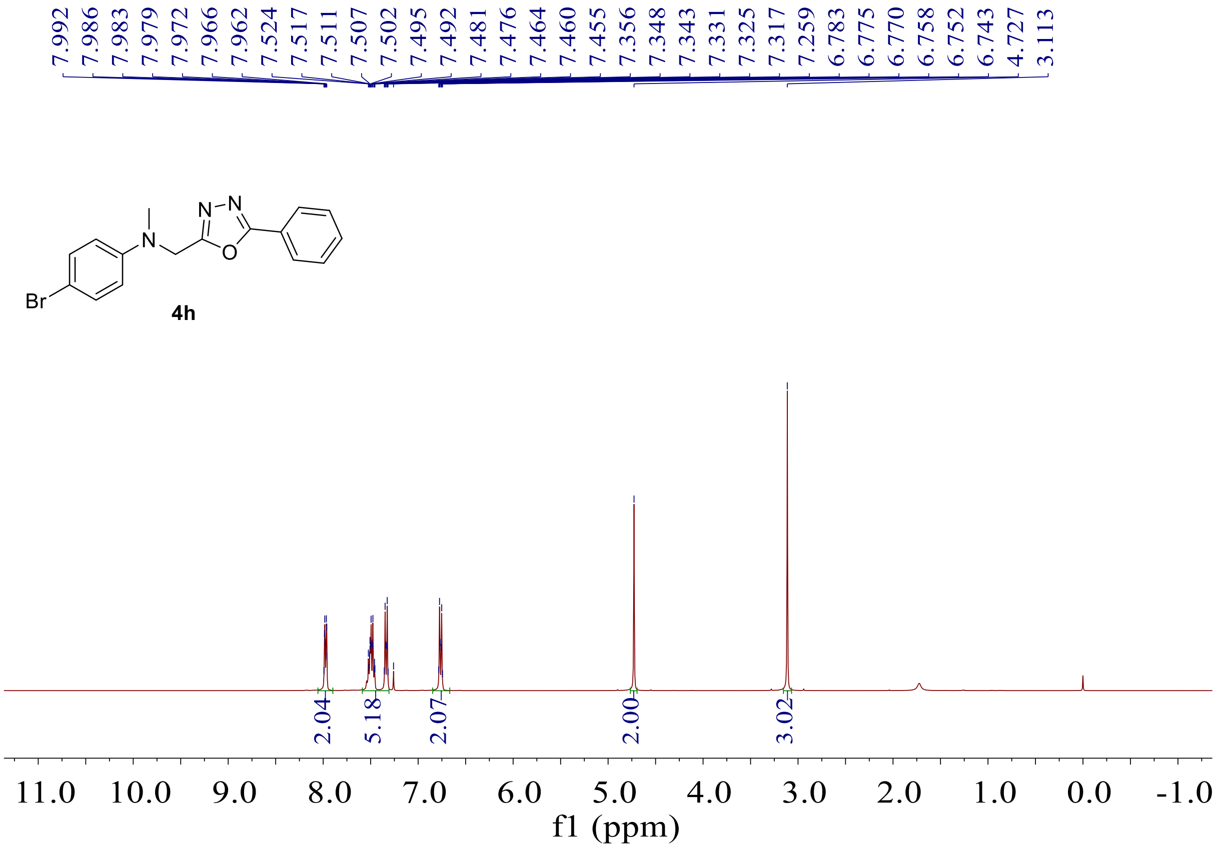

^{13}C{^{1}H} NMR (100 MHz, $CDCl_3$) of compound **4h**

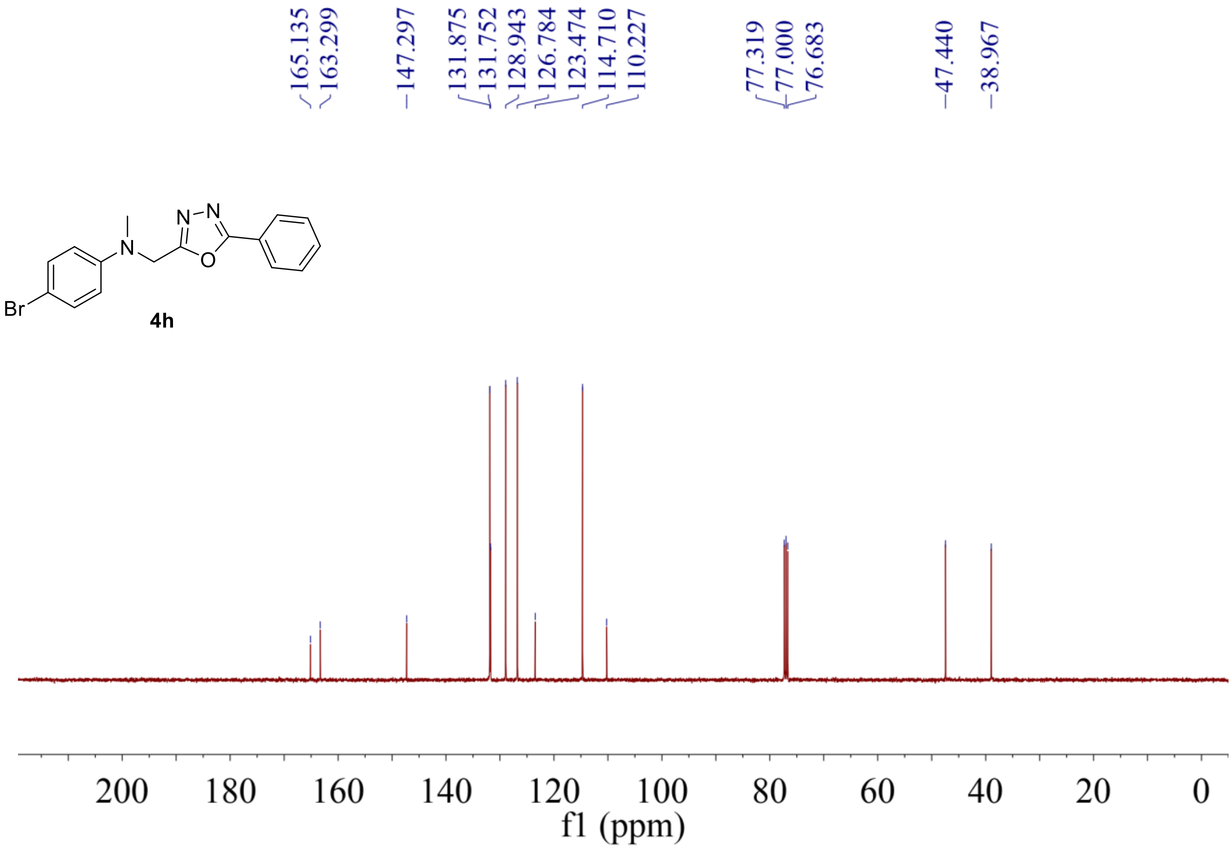

^{1}H NMR (400 MHz, $CDCl_3$) of compound **4i**

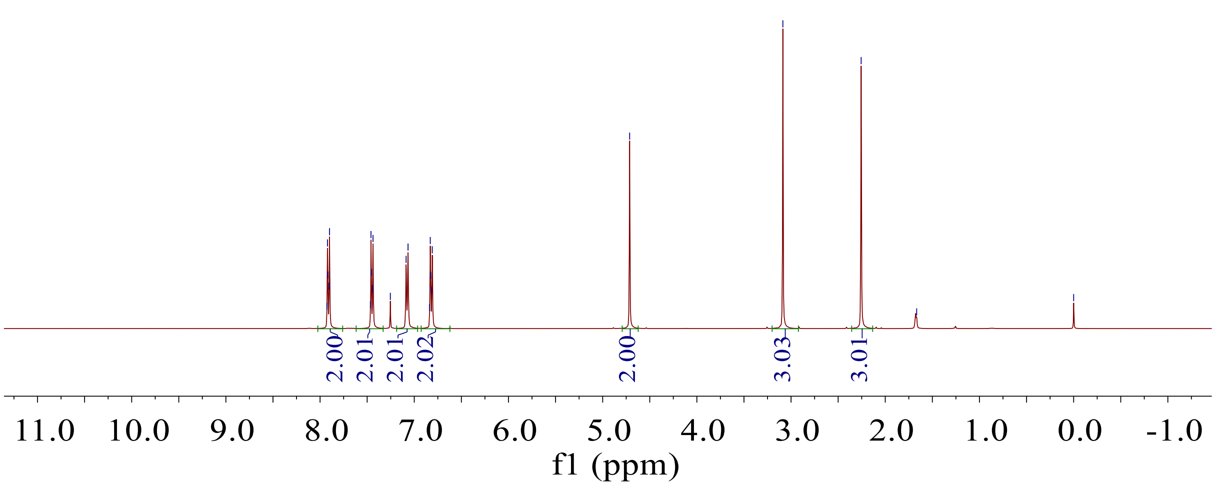

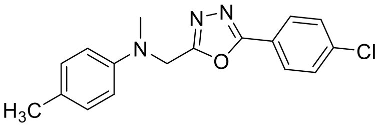

4i

$^{13}C\{^{1}H\}$ NMR (100 MHz, $CDCl_3$) of compound **4i**

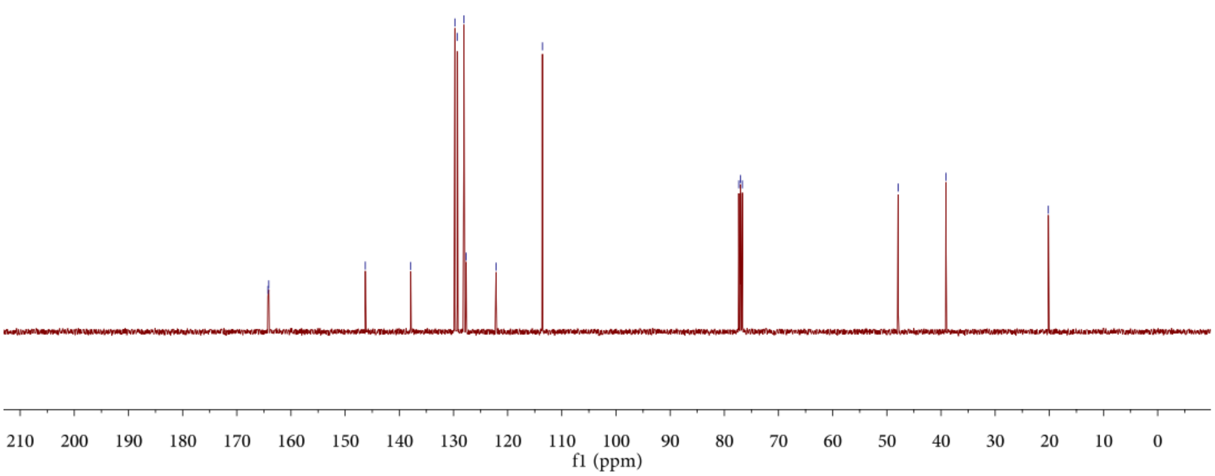

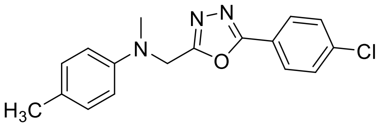

4i

^{1}H NMR (400 MHz, $CDCl_3$) of compound **4j**

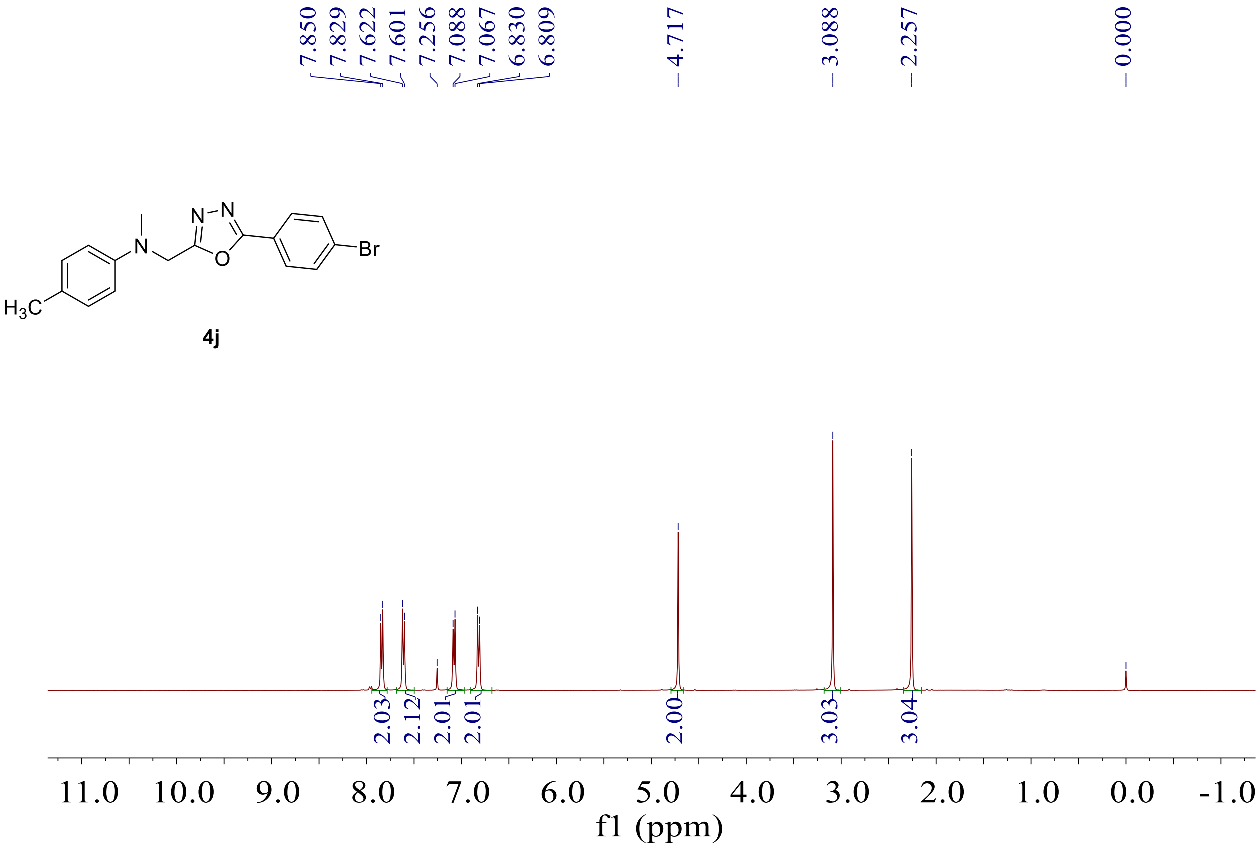

$^{13}C\{^{1}H\}$ NMR (100 MHz, $CDCl_3$) of compound **4j**

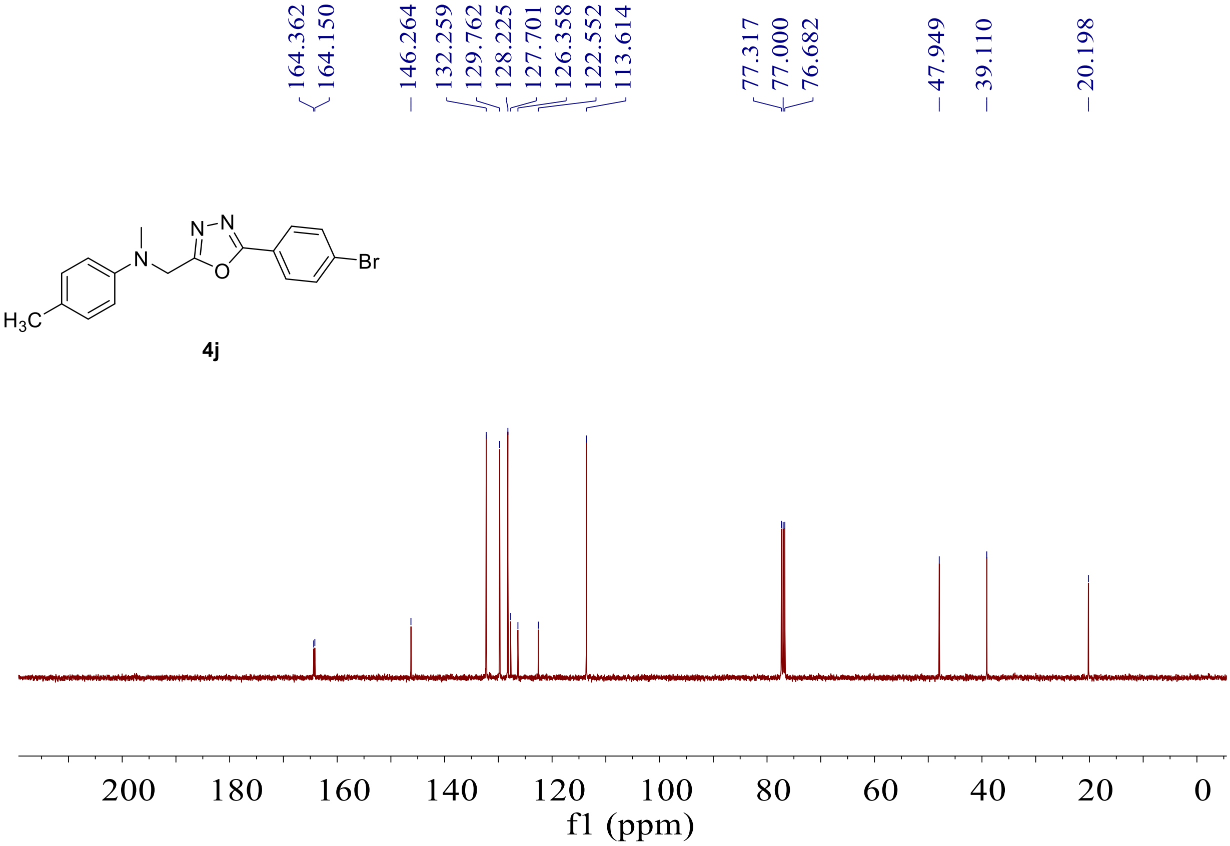

^{1}H NMR (400 MHz, $CDCl_3$) of compound **4k**

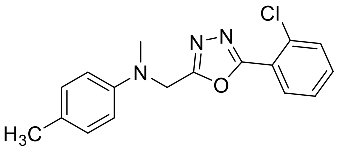

4k

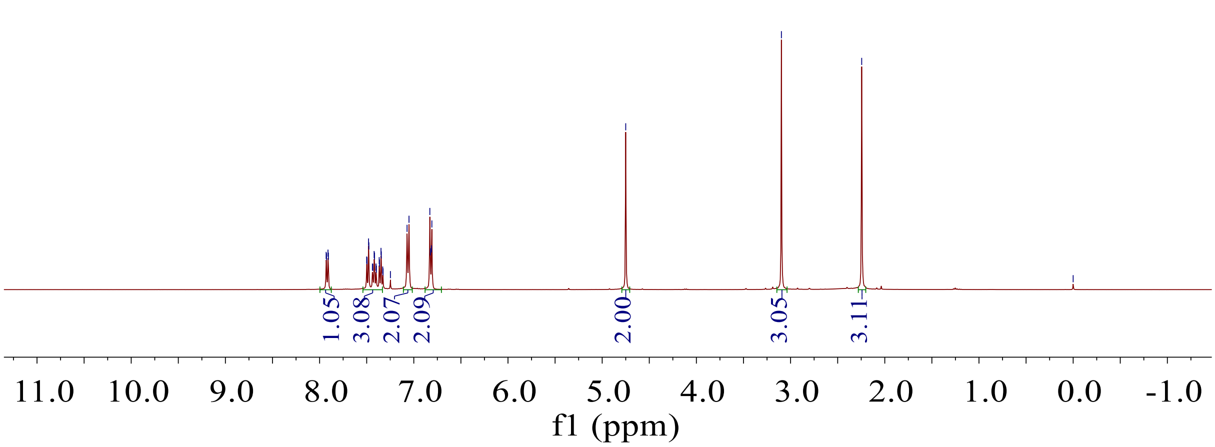

^{13}C{^{1}H} NMR (100 MHz, $CDCl_3$) of compound **4k**

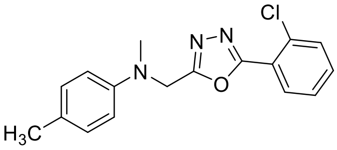

4k

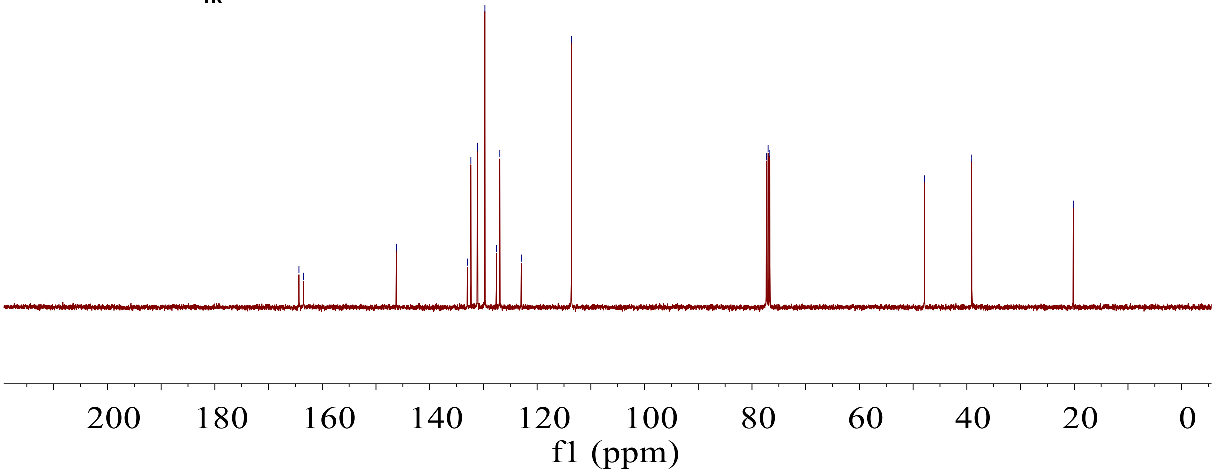

^{1}H NMR (400 MHz, $CDCl_3$) of compound **4l**

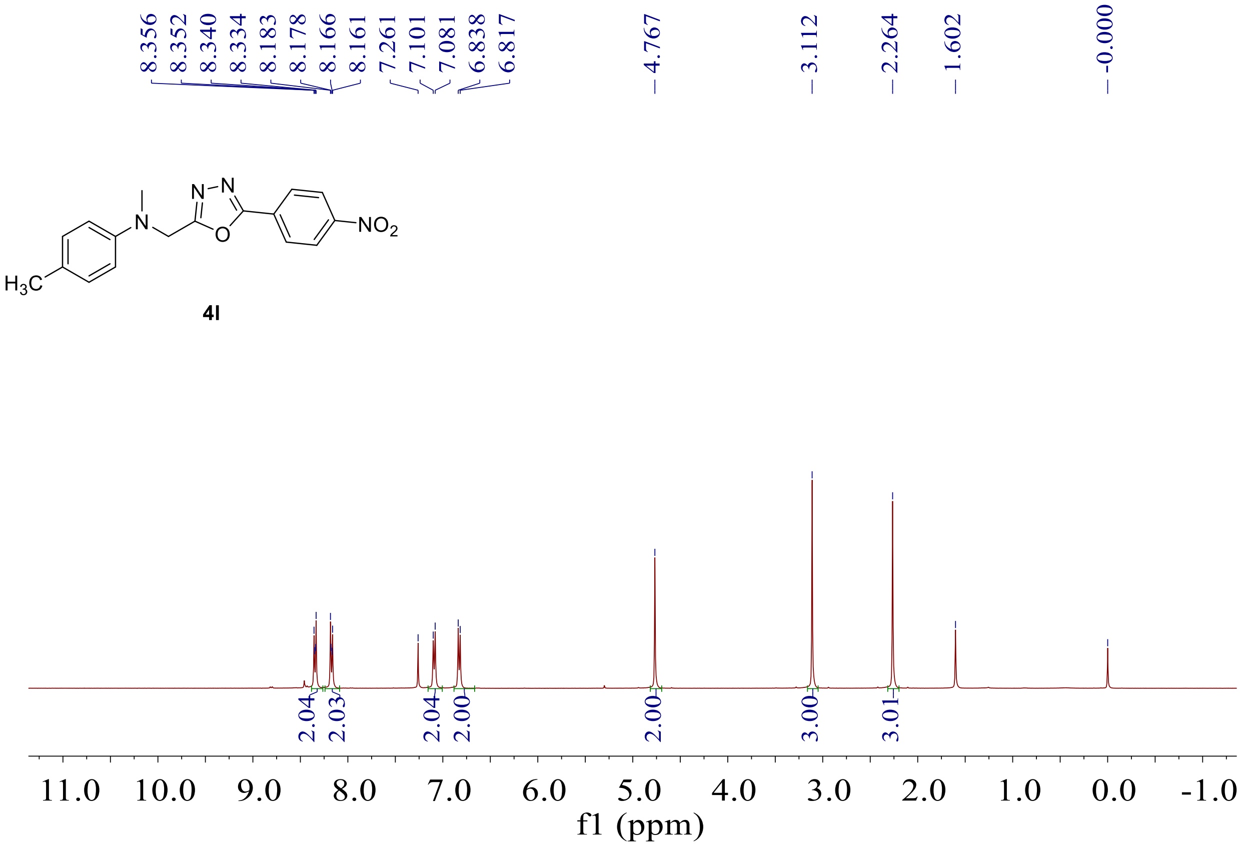

^{13}C{^{1}H} NMR (100 MHz, $CDCl_3$) of compound **4l**

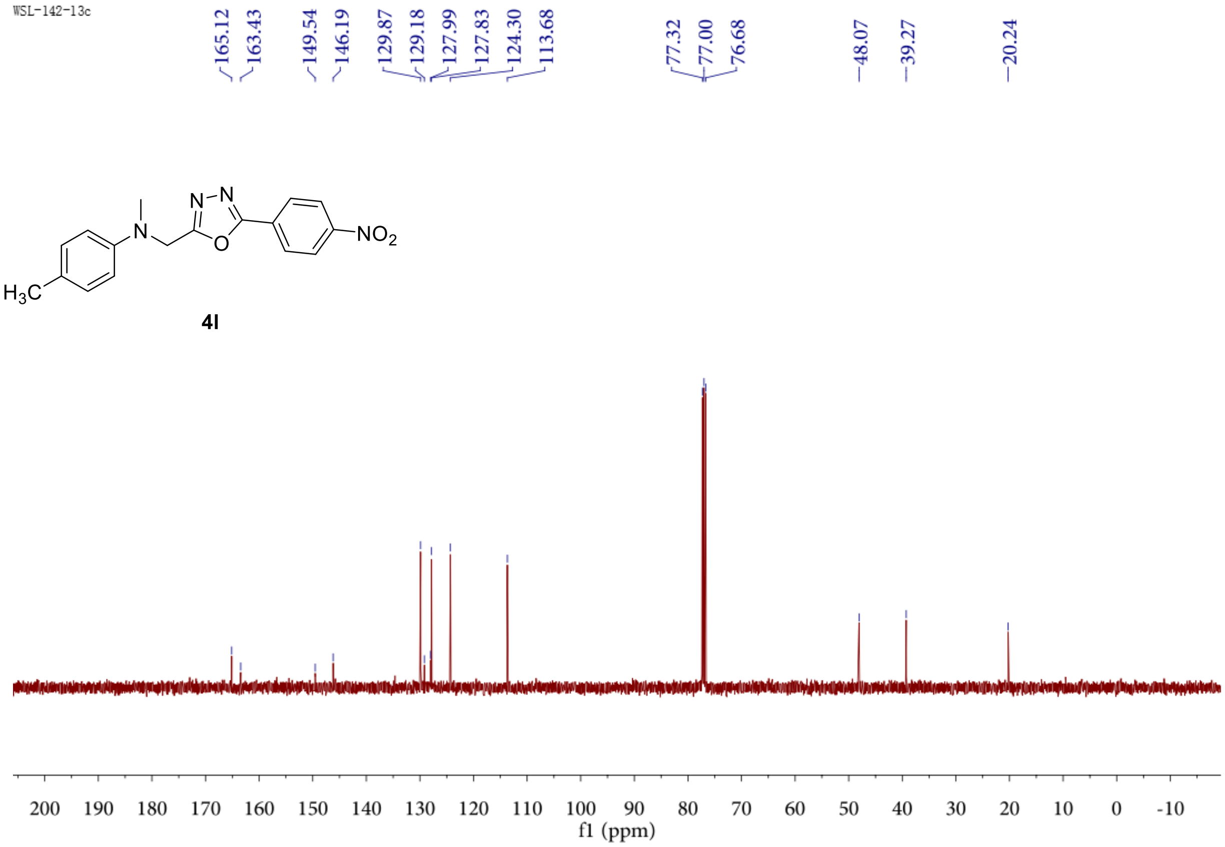

^{1}H NMR (400 MHz, $CDCl_3$) of compound **4m**

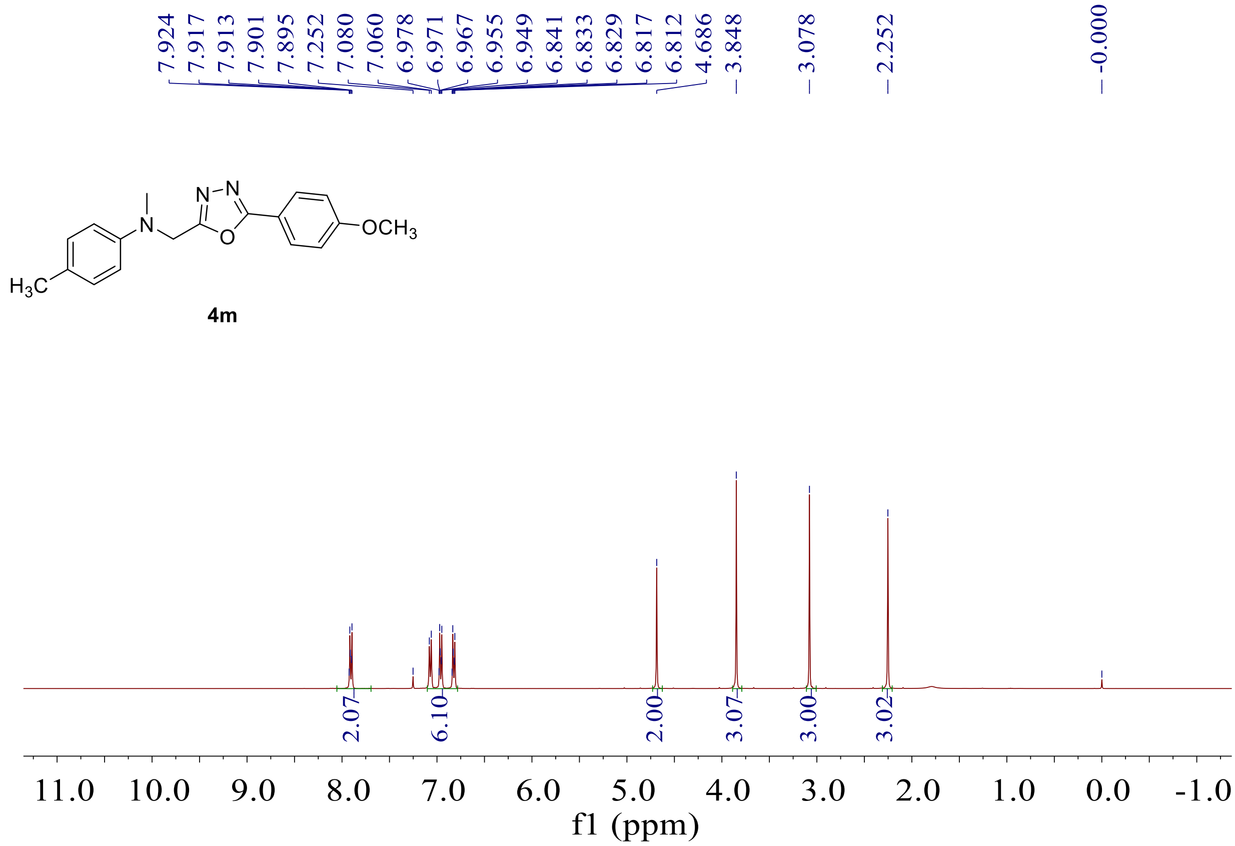

^{13}C{^{1}H} NMR (100 MHz, $CDCl_3$) of compound **4m**

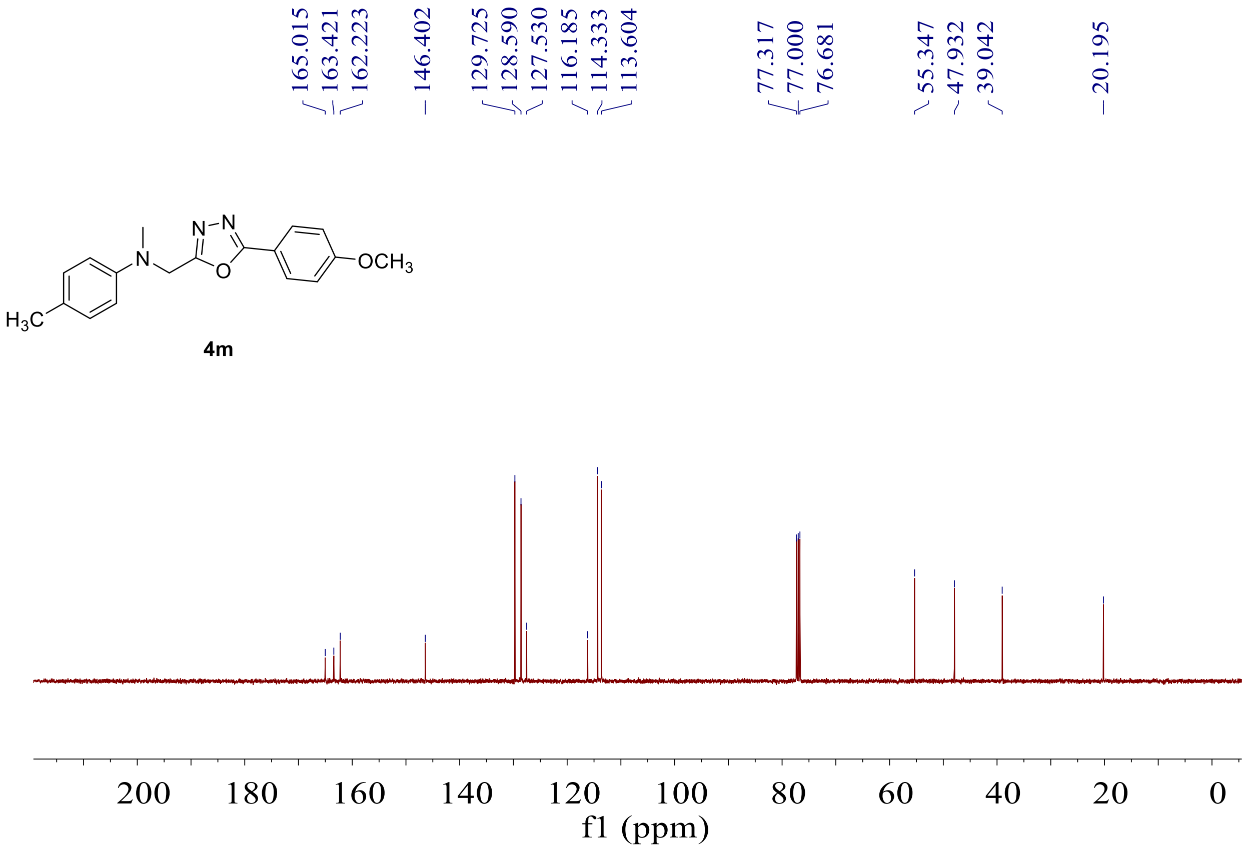

^{1}H NMR (400 MHz, $CDCl_3$) of compound **4n**

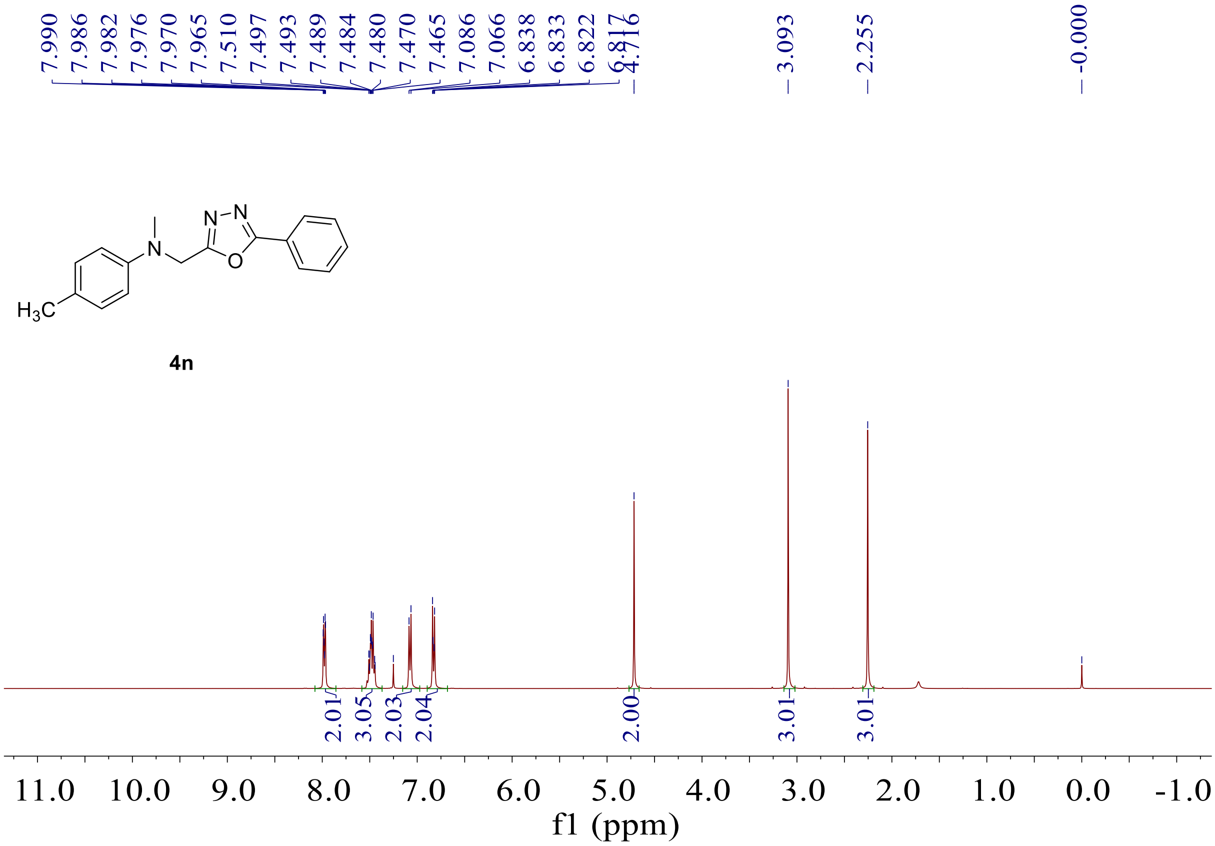

^{13}C{^{1}H} NMR (100 MHz, $CDCl_3$) of compound **4n**

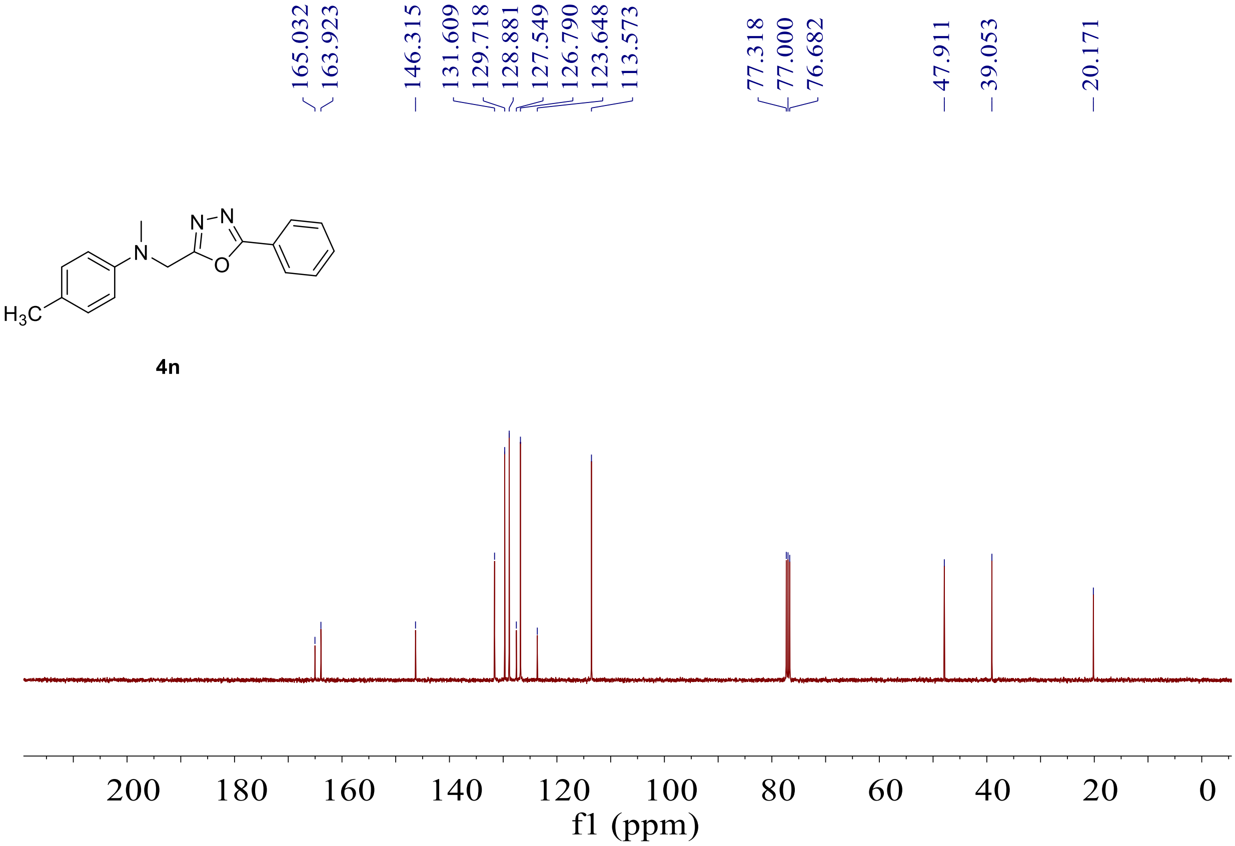

^{1}H NMR (400 MHz, $CDCl_3$) of compound **4o**

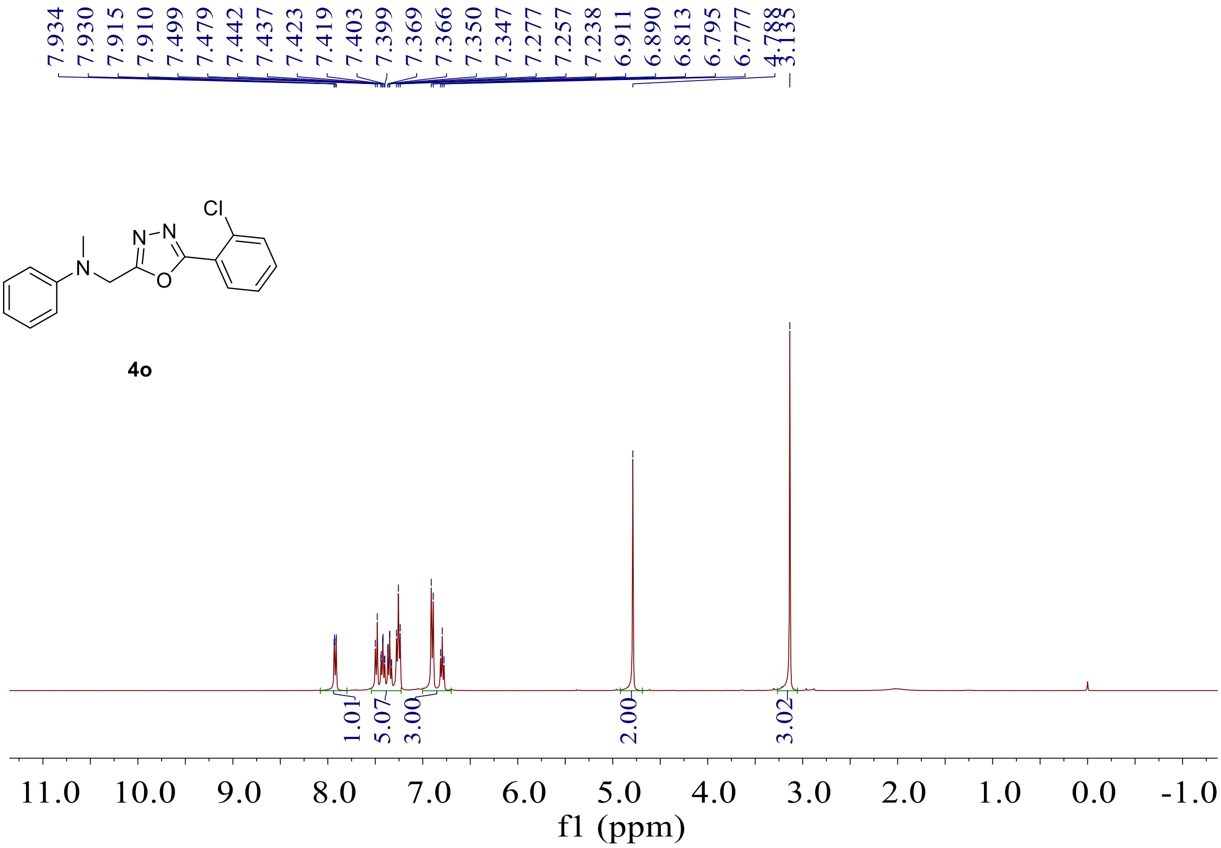

^{13}C{^{1}H} NMR (100 MHz, $CDCl_3$) of compound **4o**

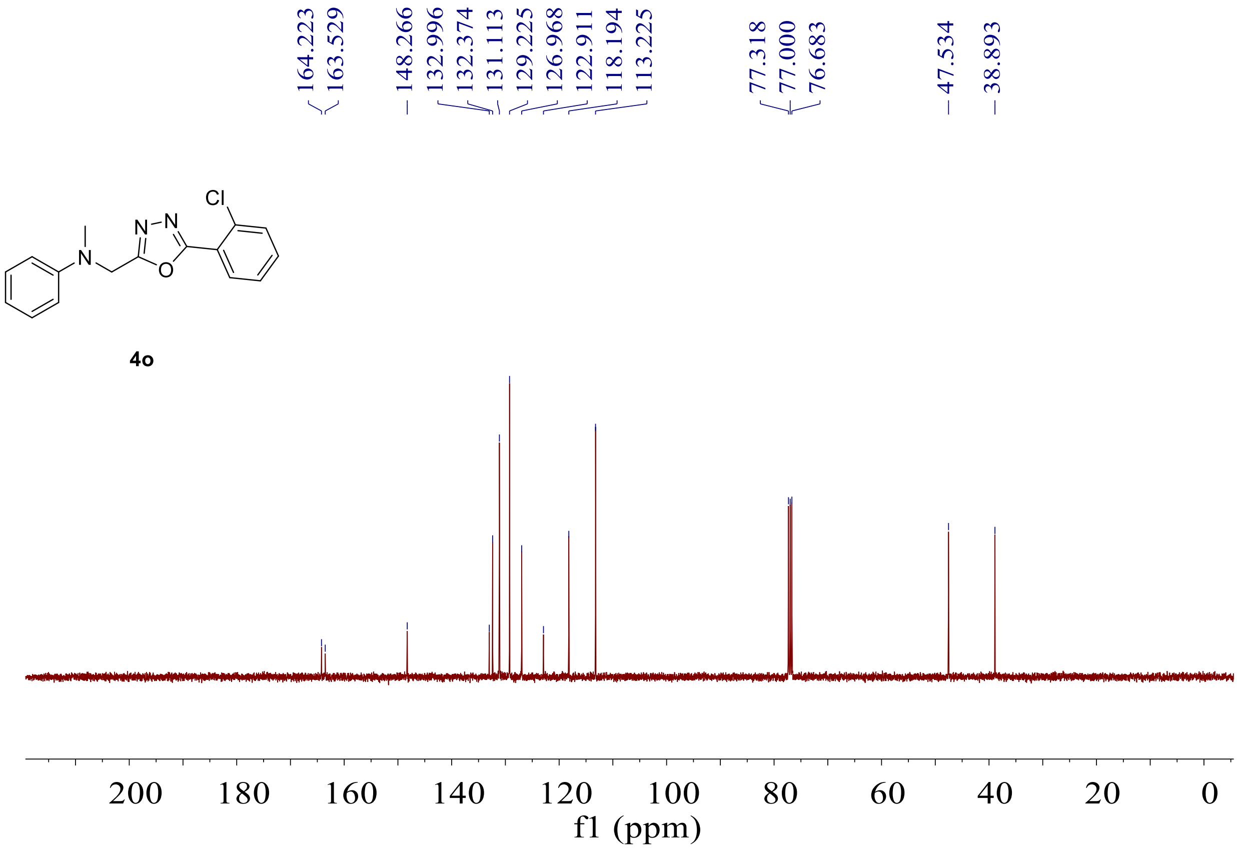

^{1}H NMR (400 MHz, $CDCl_3$) of compound **4p**

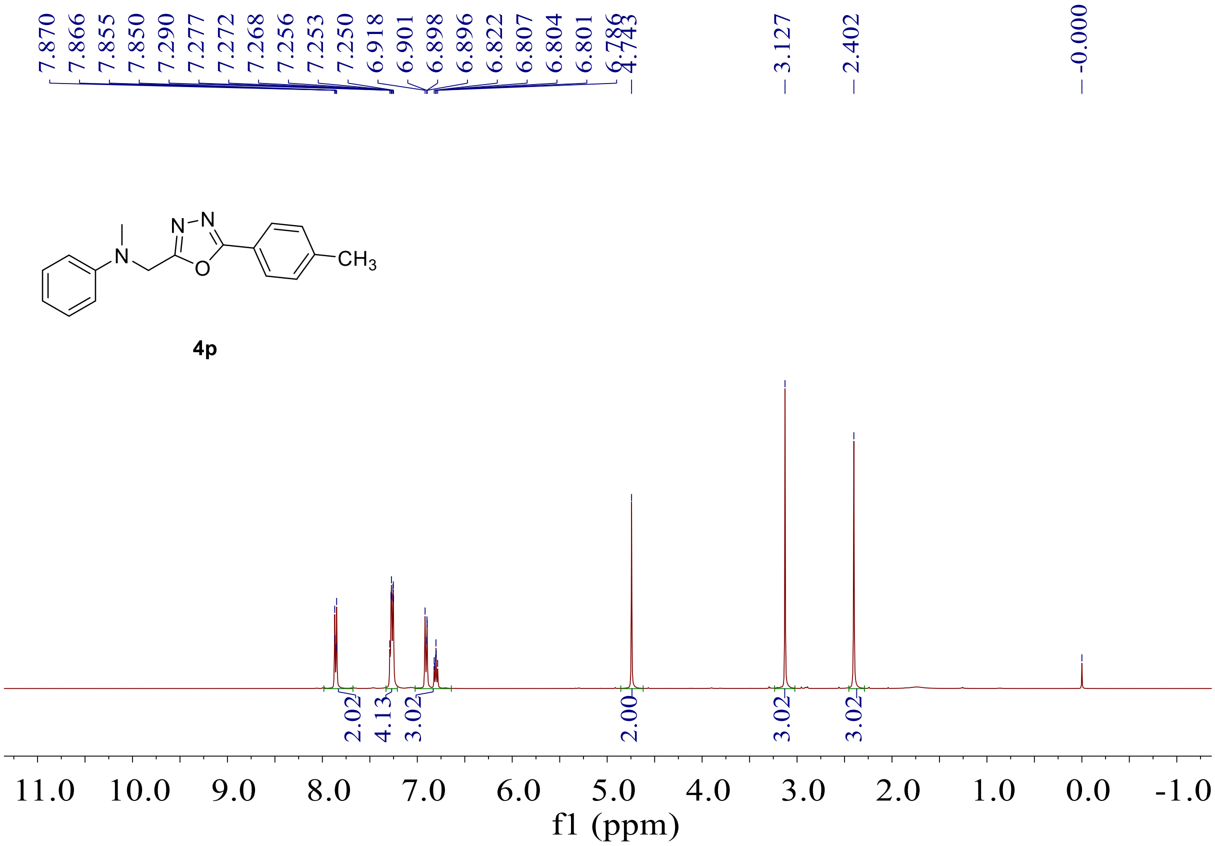

^{13}C{^{1}H} NMR (100 MHz, $CDCl_3$) of compound **4p**

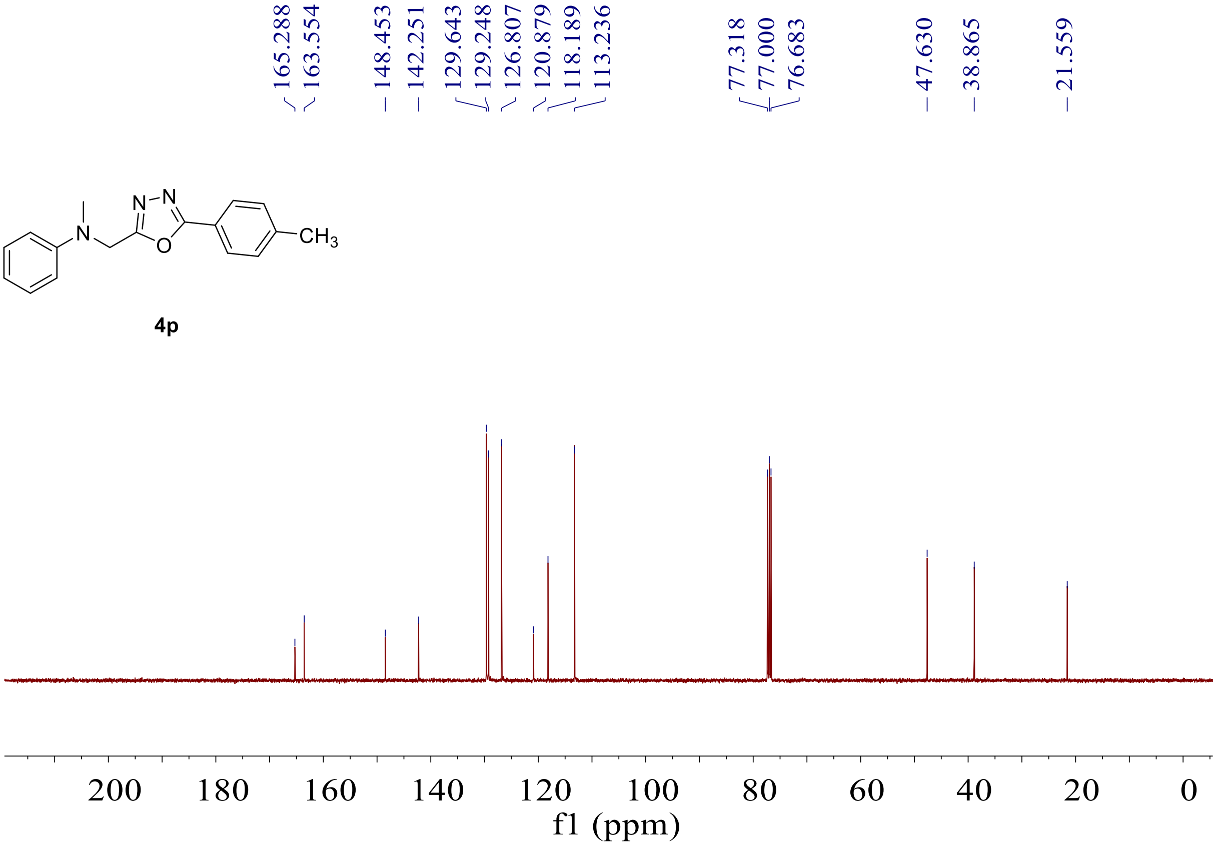

^{1}H NMR (400 MHz, $CDCl_3$) of compound **4q**

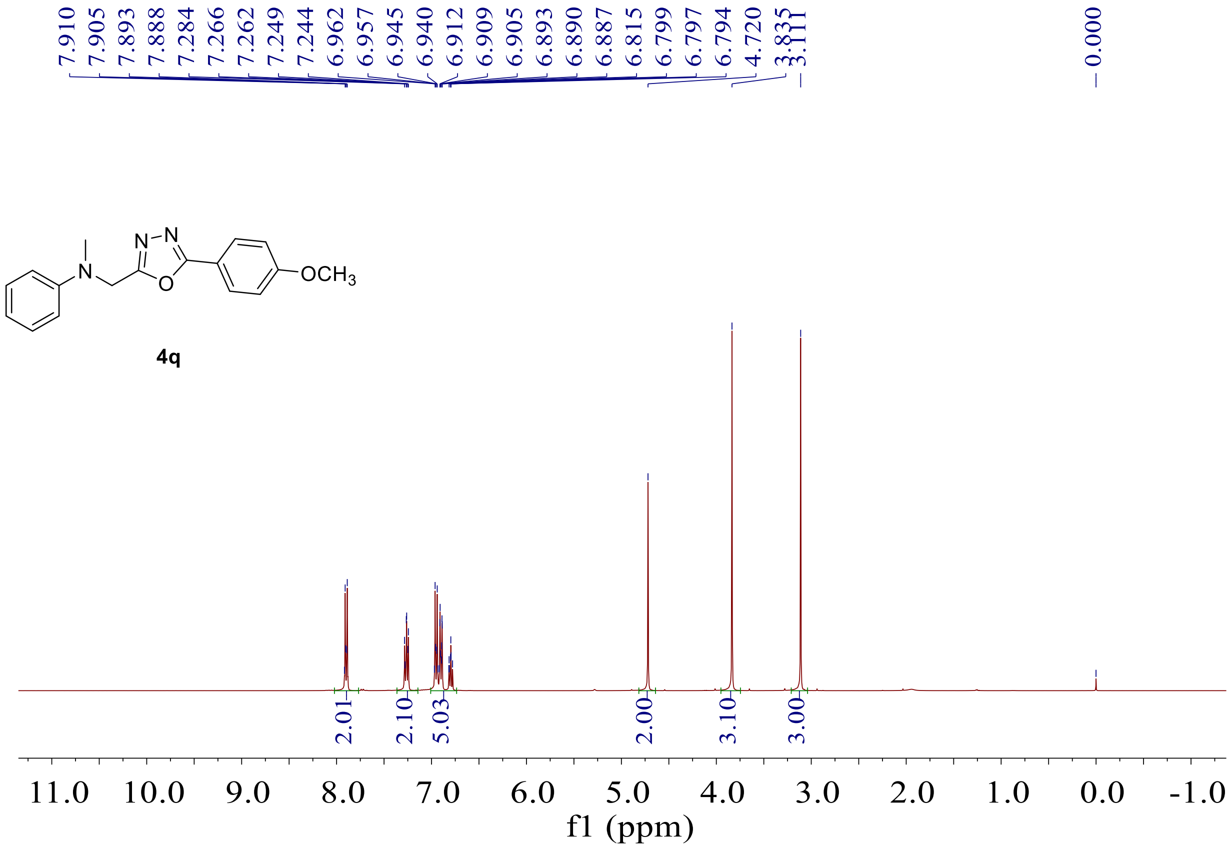

^{13}C{^{1}H} NMR (100 MHz, $CDCl_3$) of compound **4q**

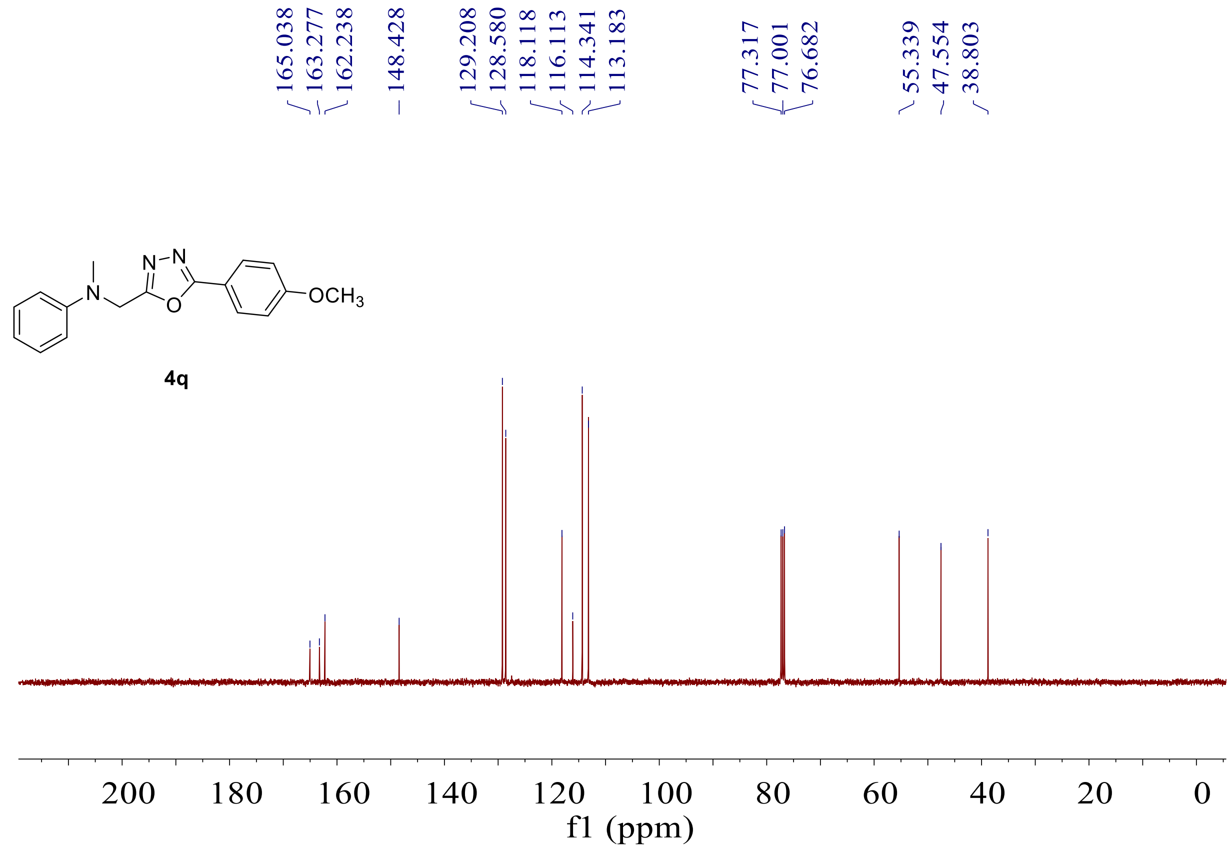

^{1}H NMR (400 MHz, $CDCl_3$) of compound **4r**

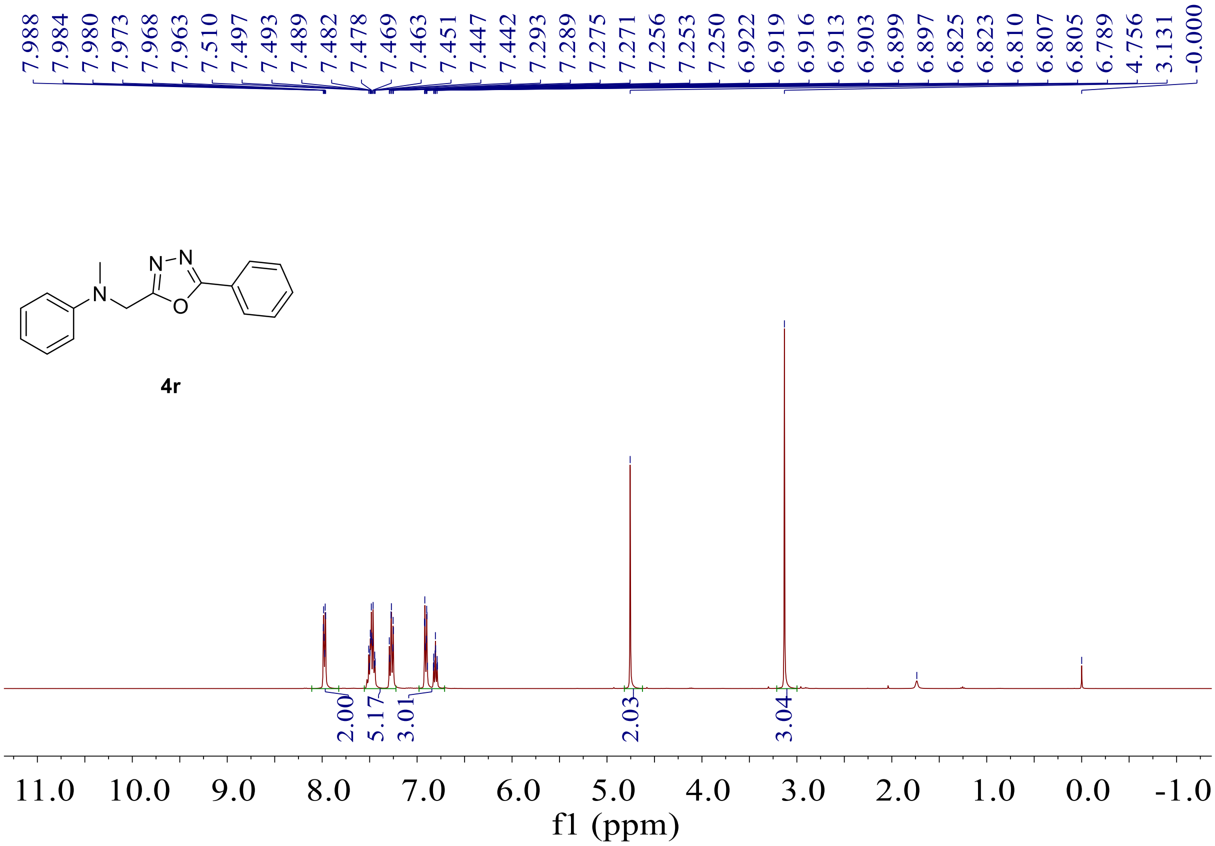

^{13}C{^{1}H} NMR (100 MHz, $CDCl_3$) of compound **4r**

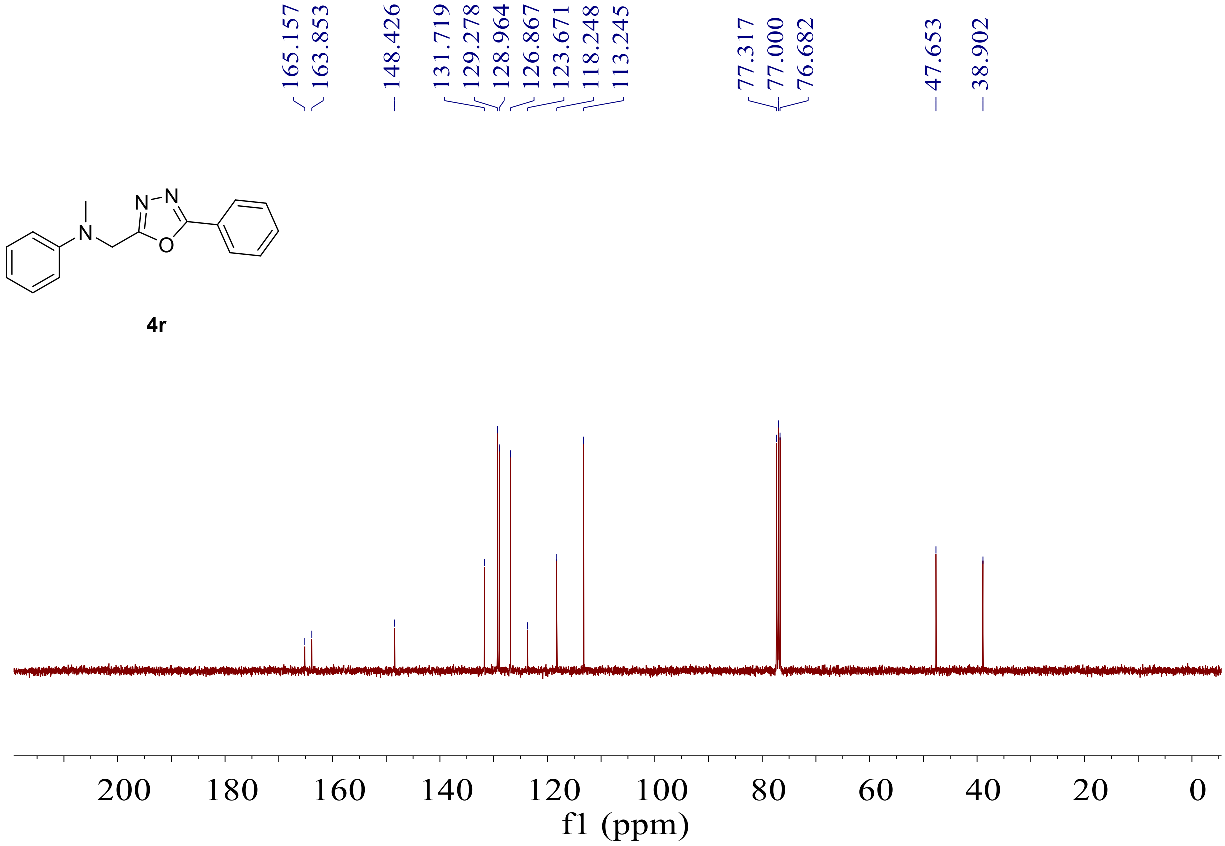

^{1}H NMR (400 MHz, $CDCl_3$) of compound **4s**

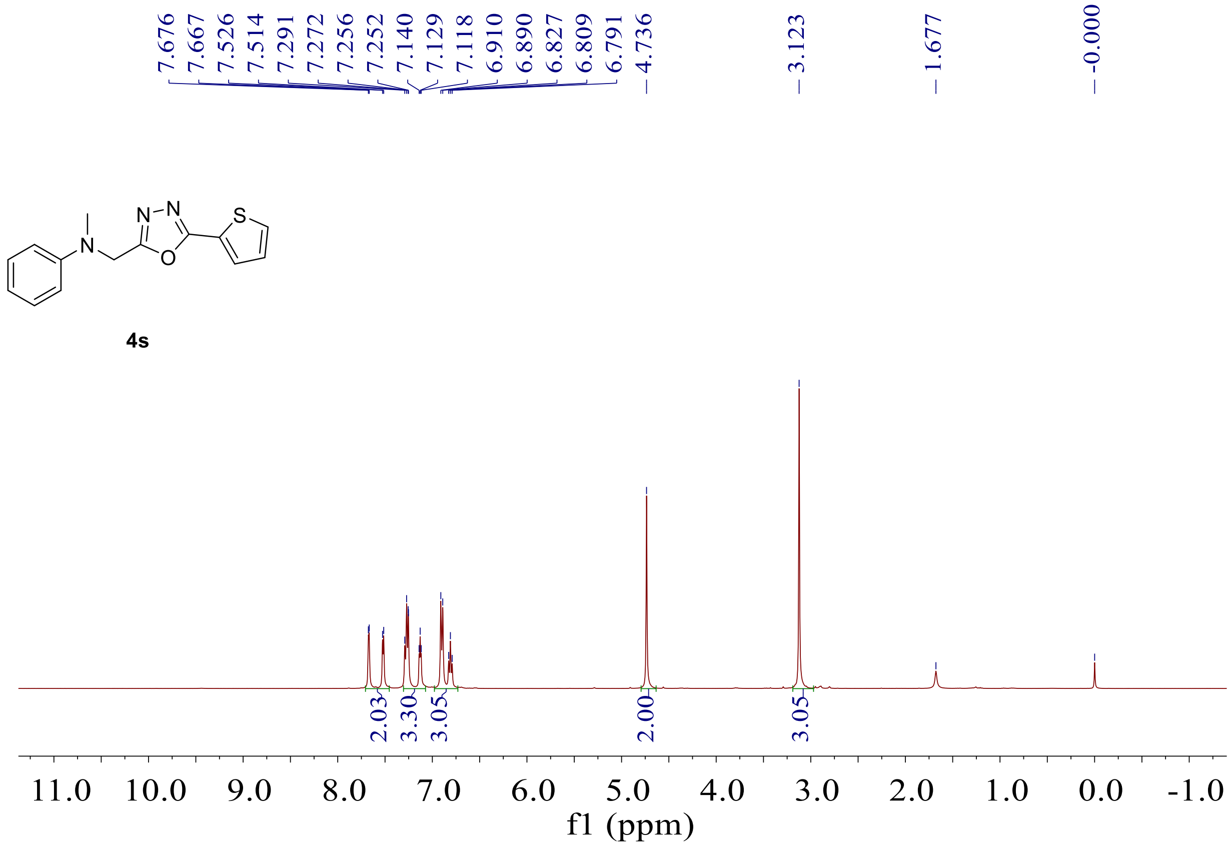

^{13}C{^{1}H} NMR (100 MHz, $CDCl_3$) of compound **4s**

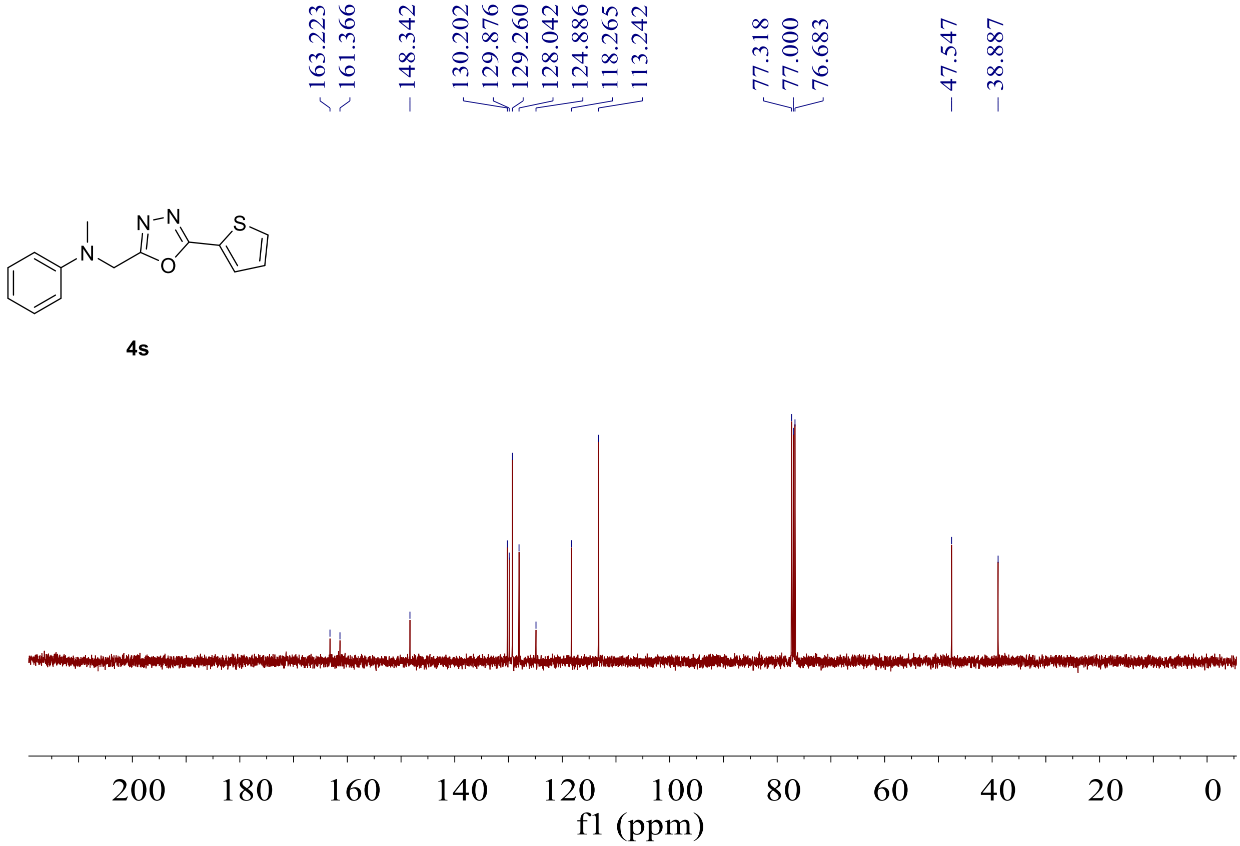

^{1}H NMR (400 MHz, $CDCl_3$) of compound **4t**

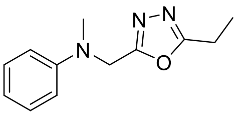

4t

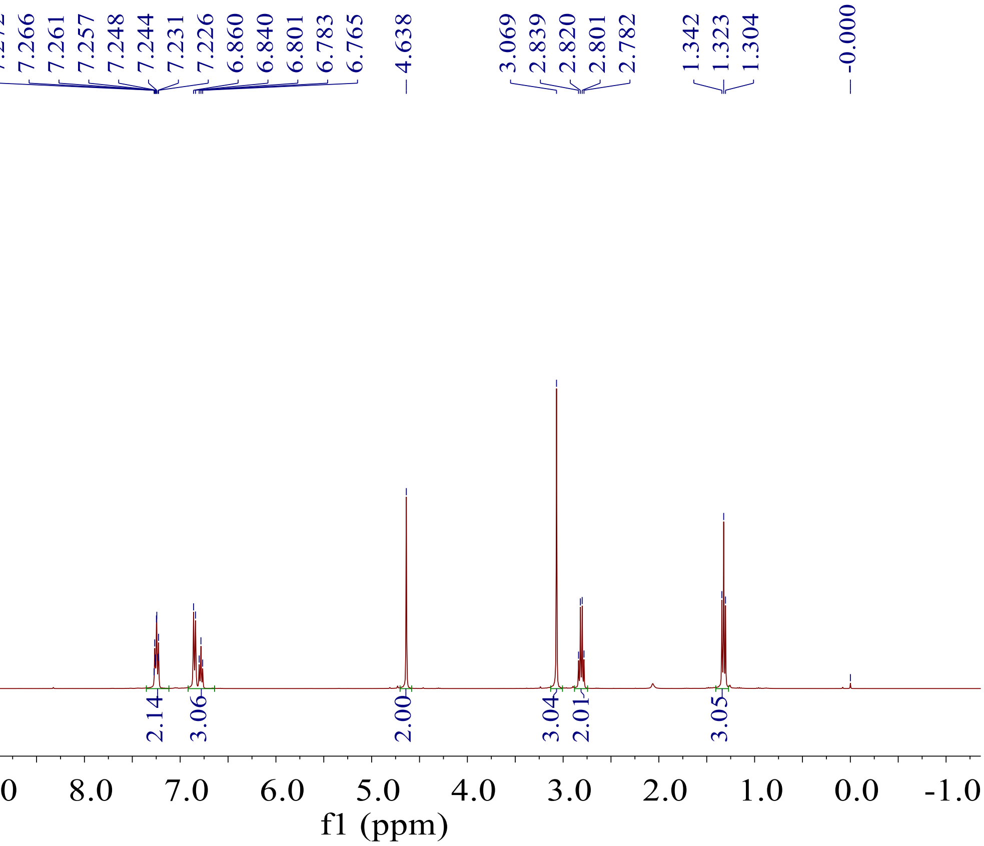

^{13}C{^{1}H} NMR (100 MHz, $CDCl_3$) of compound **4t**

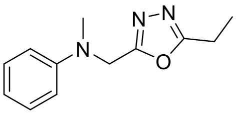

4t

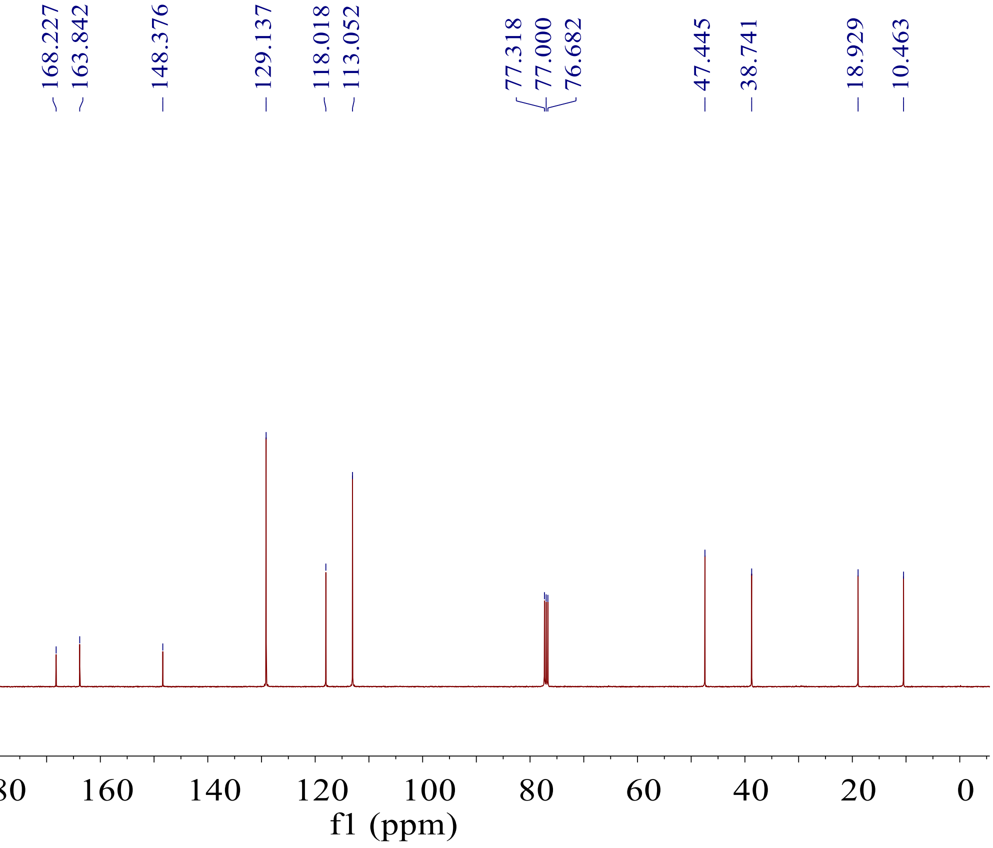

^{1}H NMR (400 MHz, $CDCl_3$) of compound **4u**

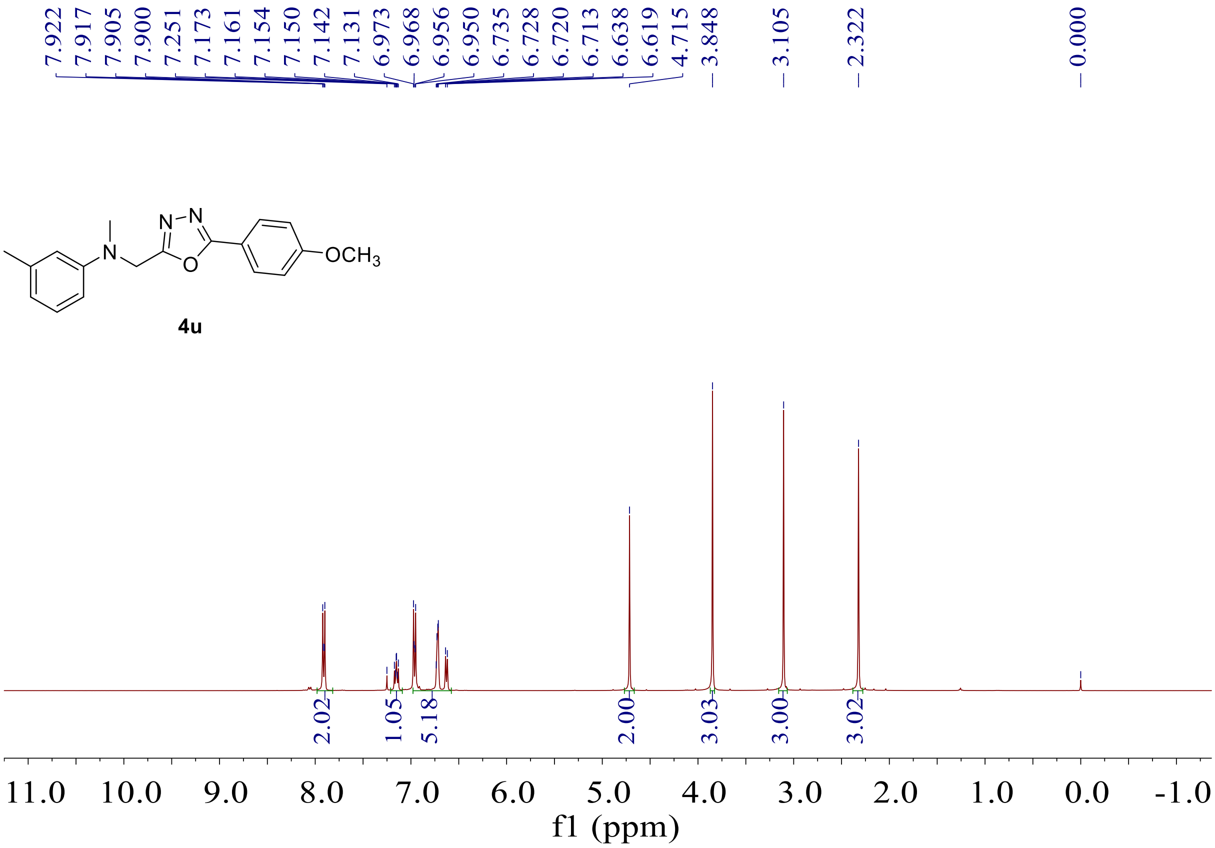

^{13}C{^{1}H} NMR (100 MHz, $CDCl_3$) of compound **4u**

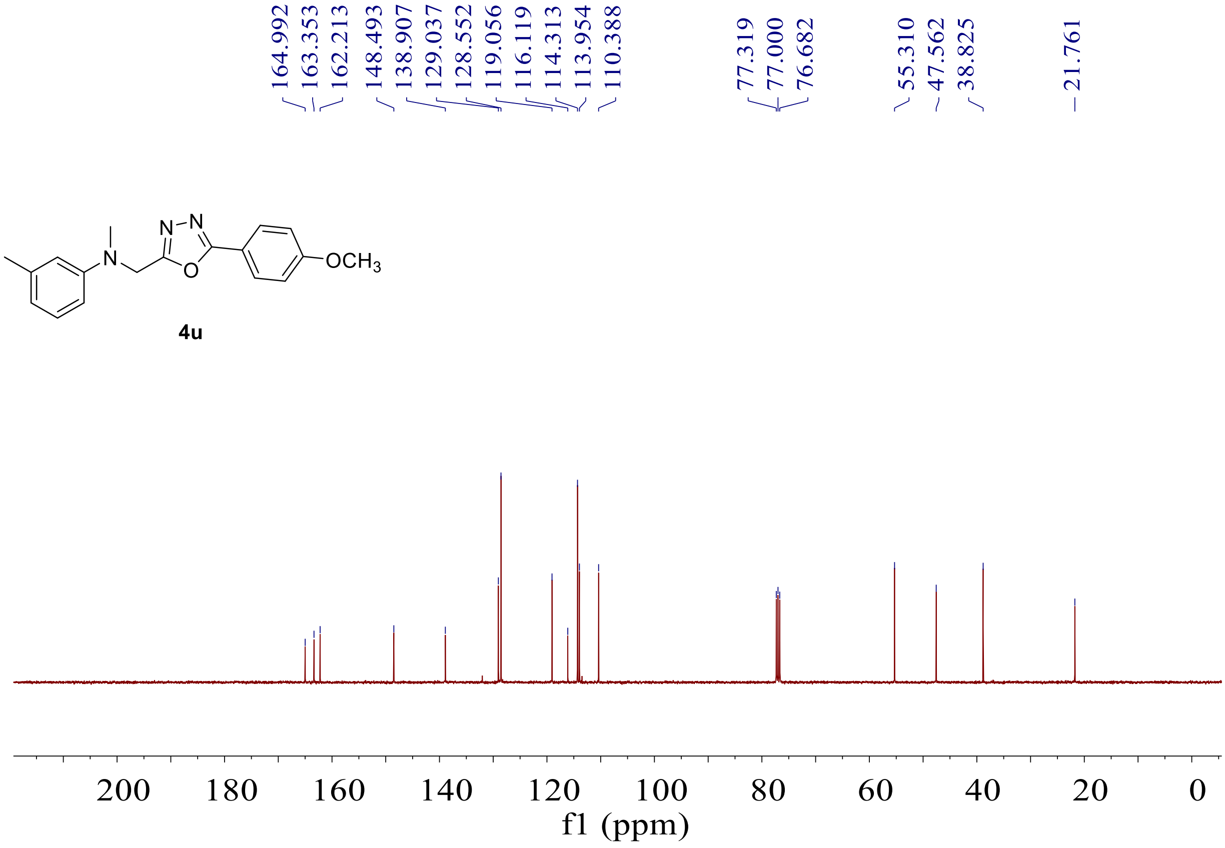

^{1}H NMR (400 MHz, $CDCl_3$) of compound **4v**

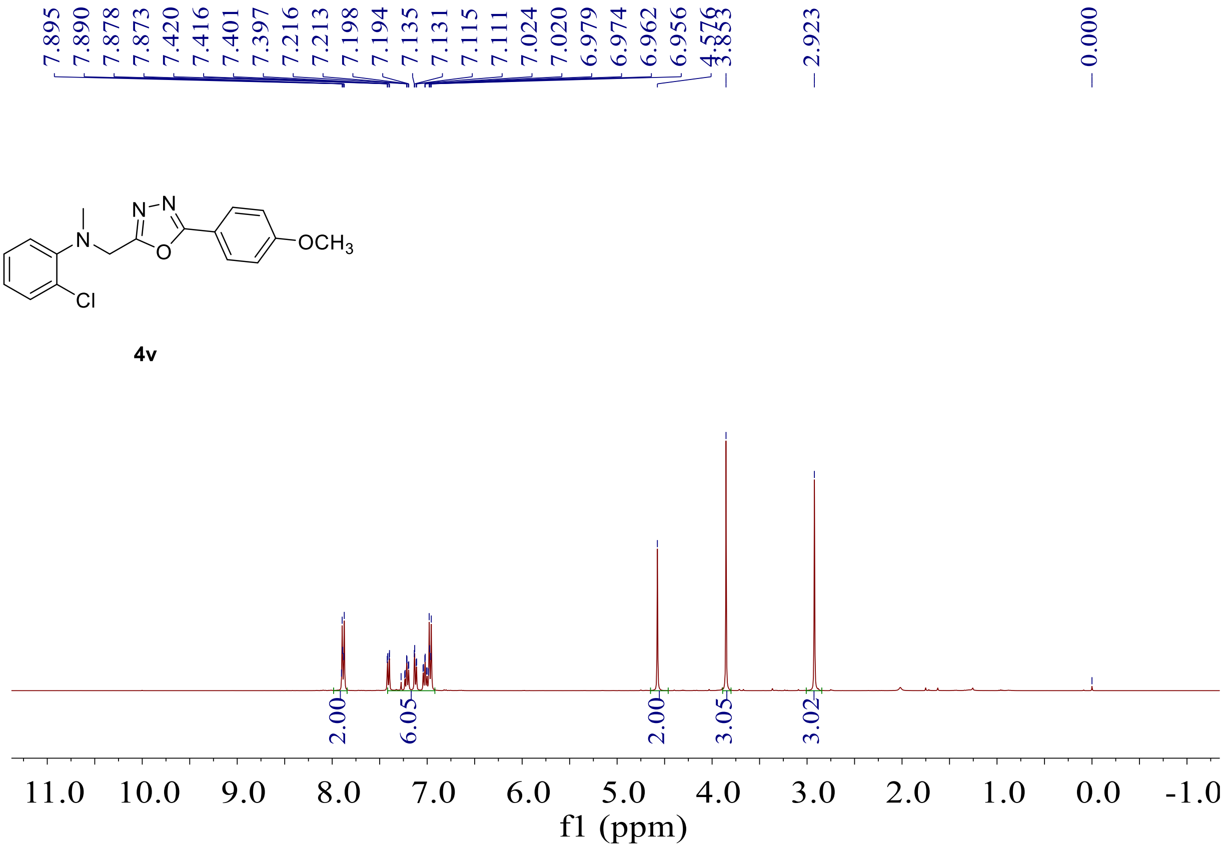

^{13}C{^{1}H} NMR (100 MHz, $CDCl_3$) of compound **4v**

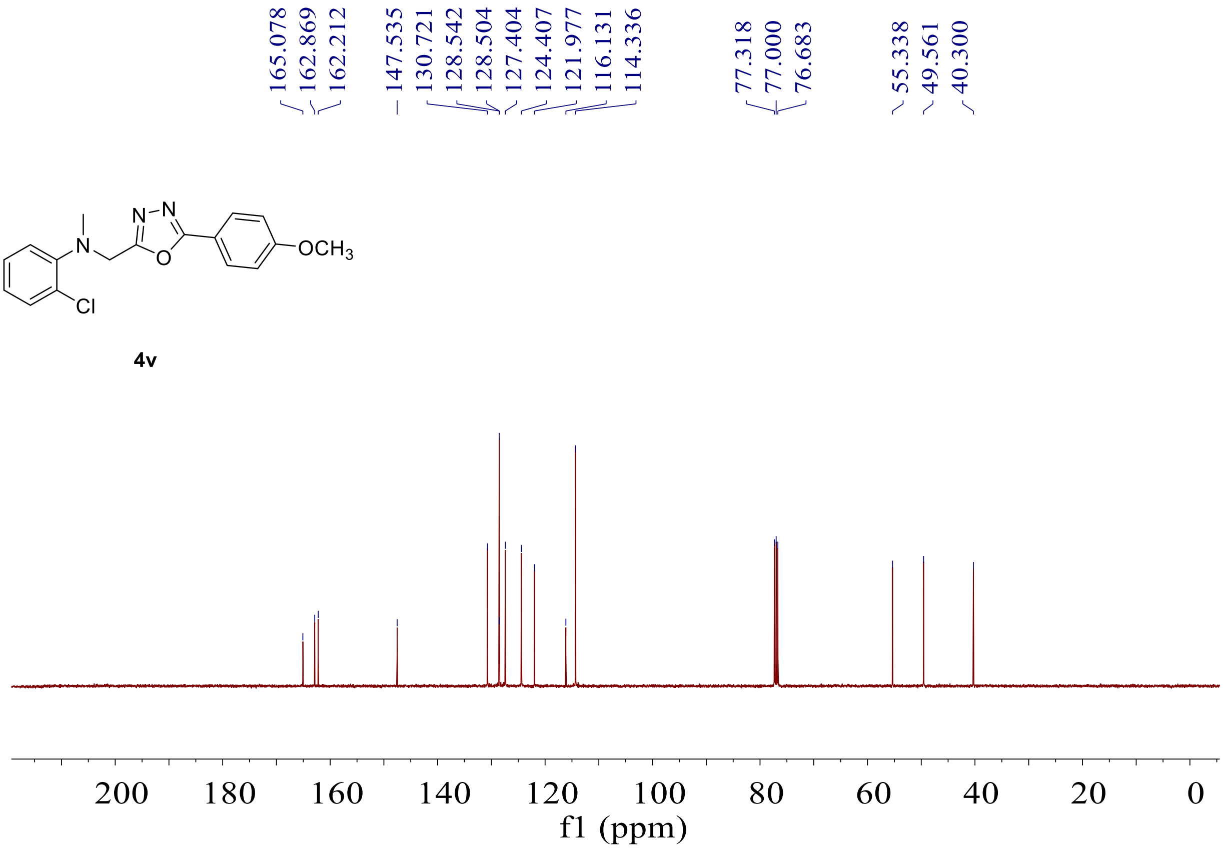

^{1}H NMR (400 MHz, $CDCl_3$) of compound **4w**

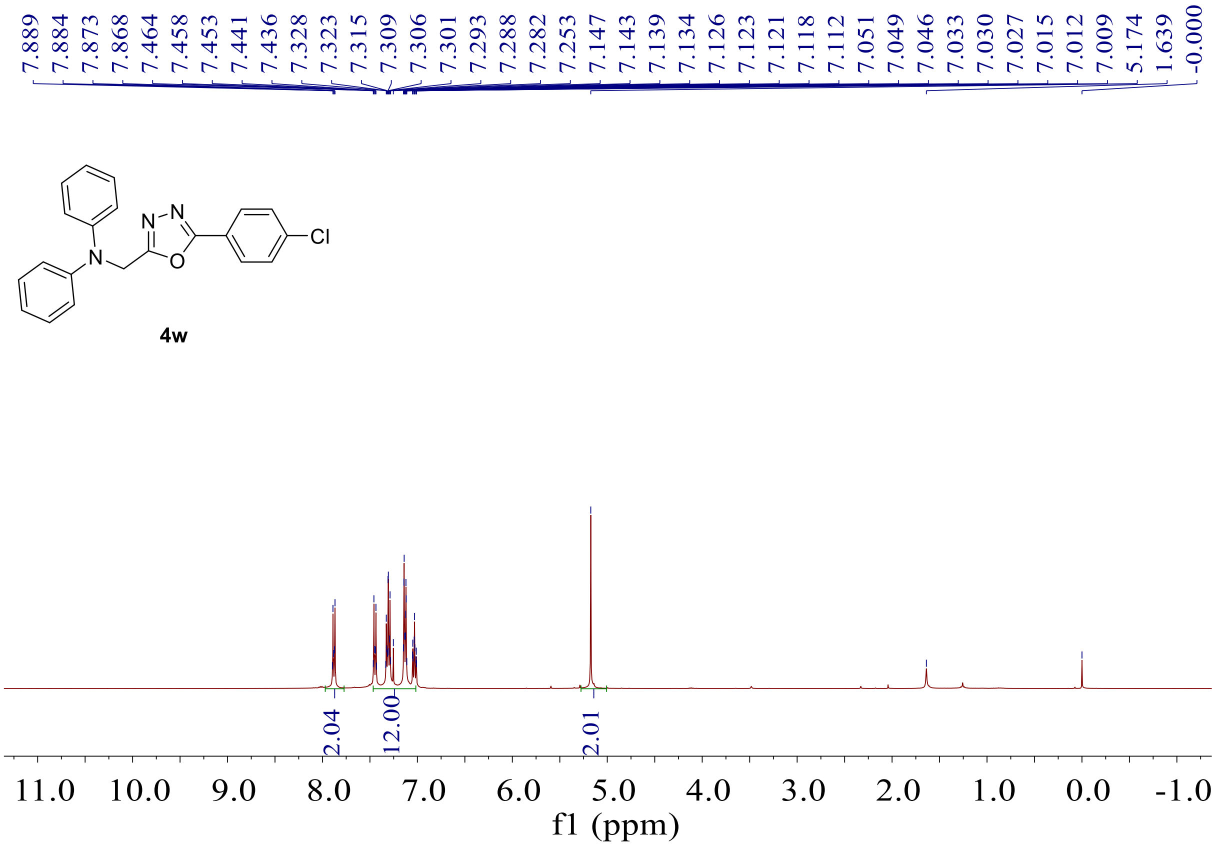

^{13}C{^{1}H} NMR (100 MHz, $CDCl_3$) of compound **4w**

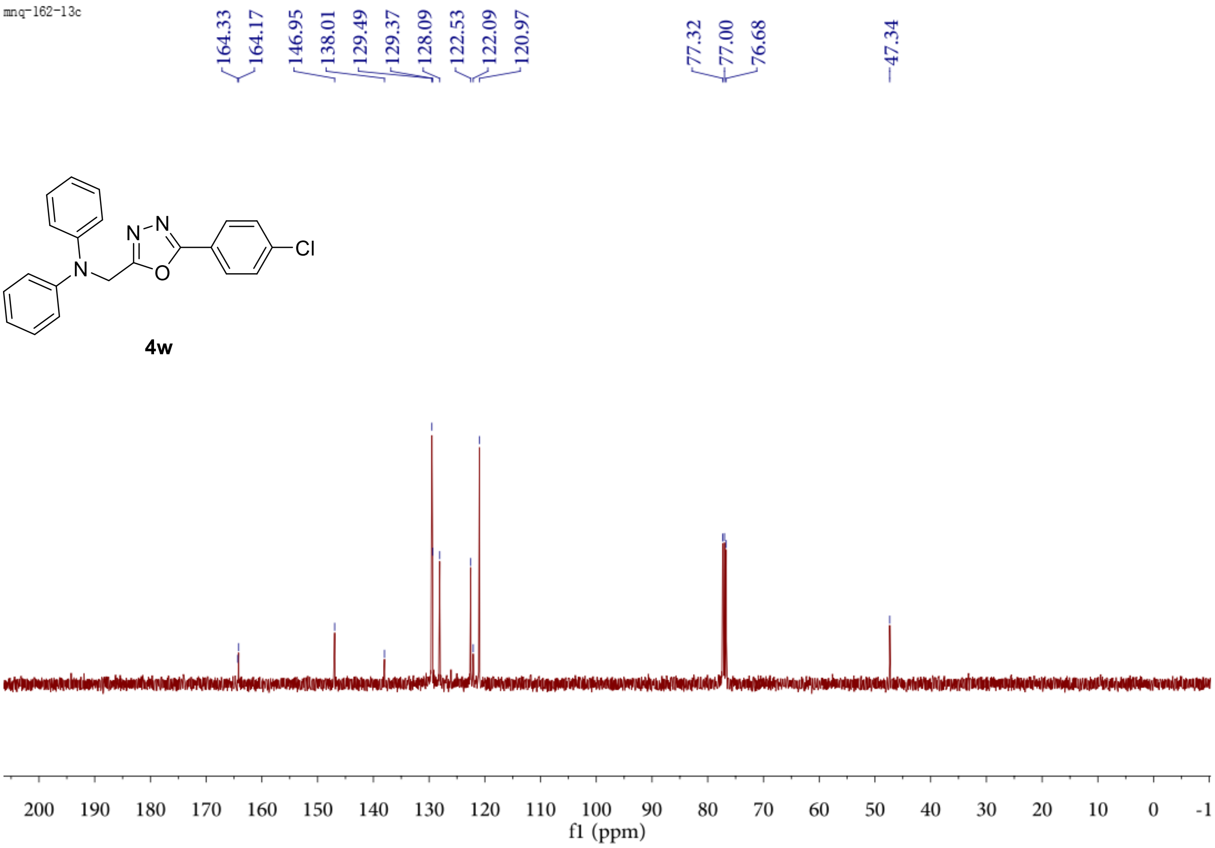

^{1}H NMR (400 MHz, $CDCl_3$) of compound **4x**

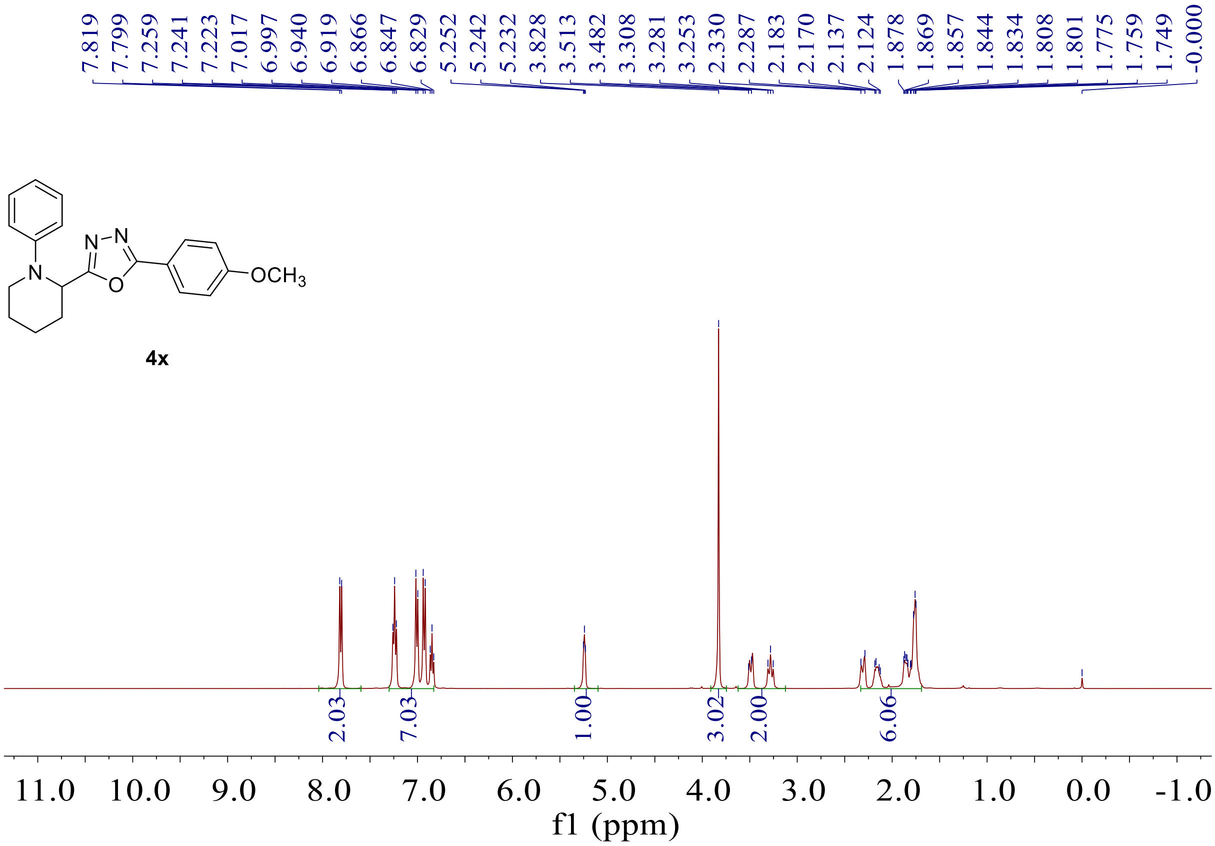

$^{13}C\{^{1}H\}$ NMR (100 MHz, $CDCl_3$) of compound **4x**

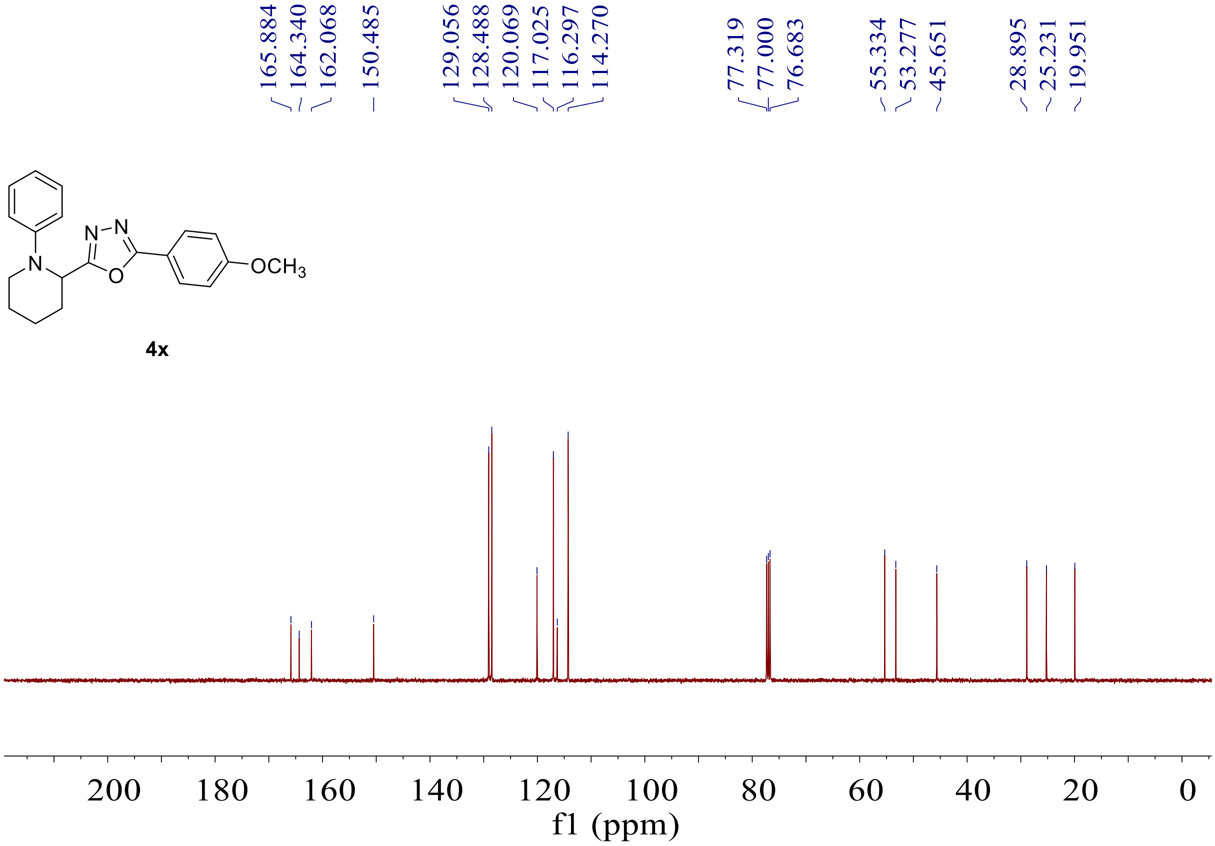

^{1}H NMR (400 MHz, $CDCl_3$) of compound **4y**

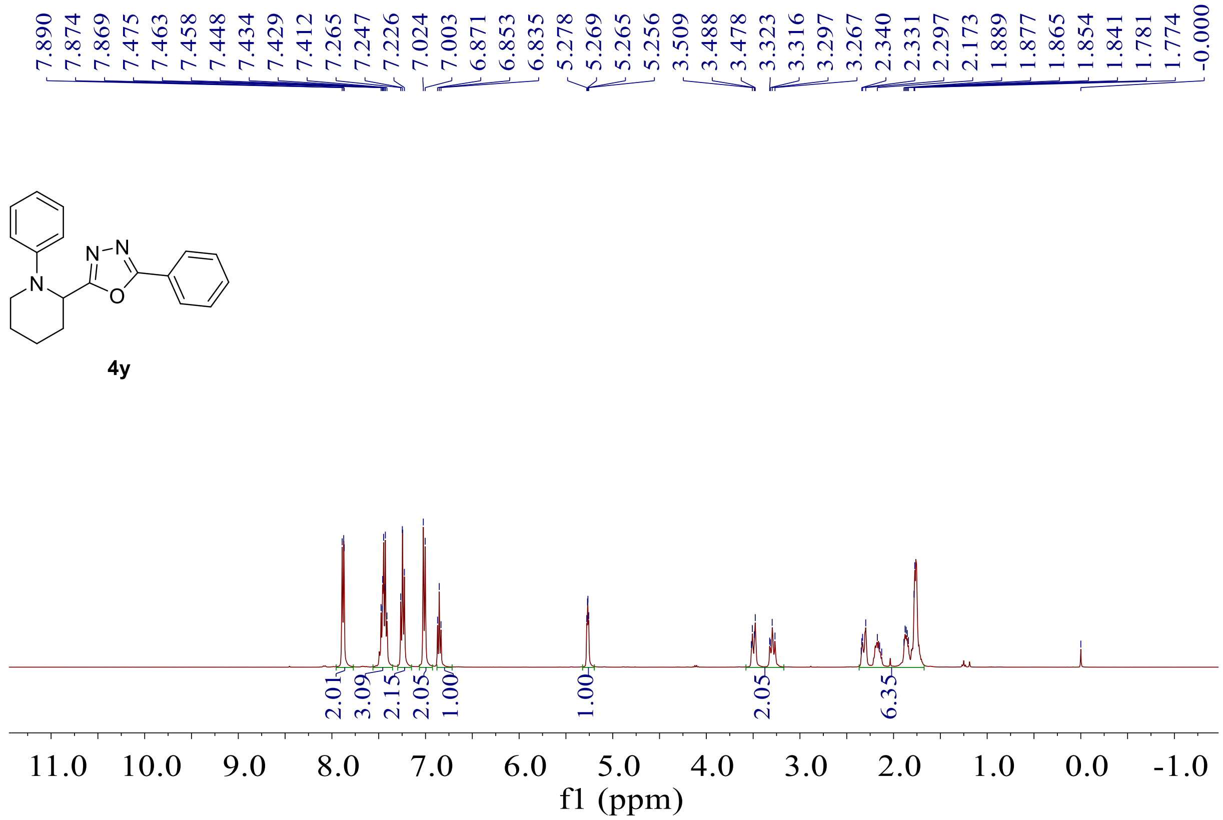

$^{13}C\{^{1}H\}$ NMR (100 MHz, $CDCl_3$) of compound **4y**

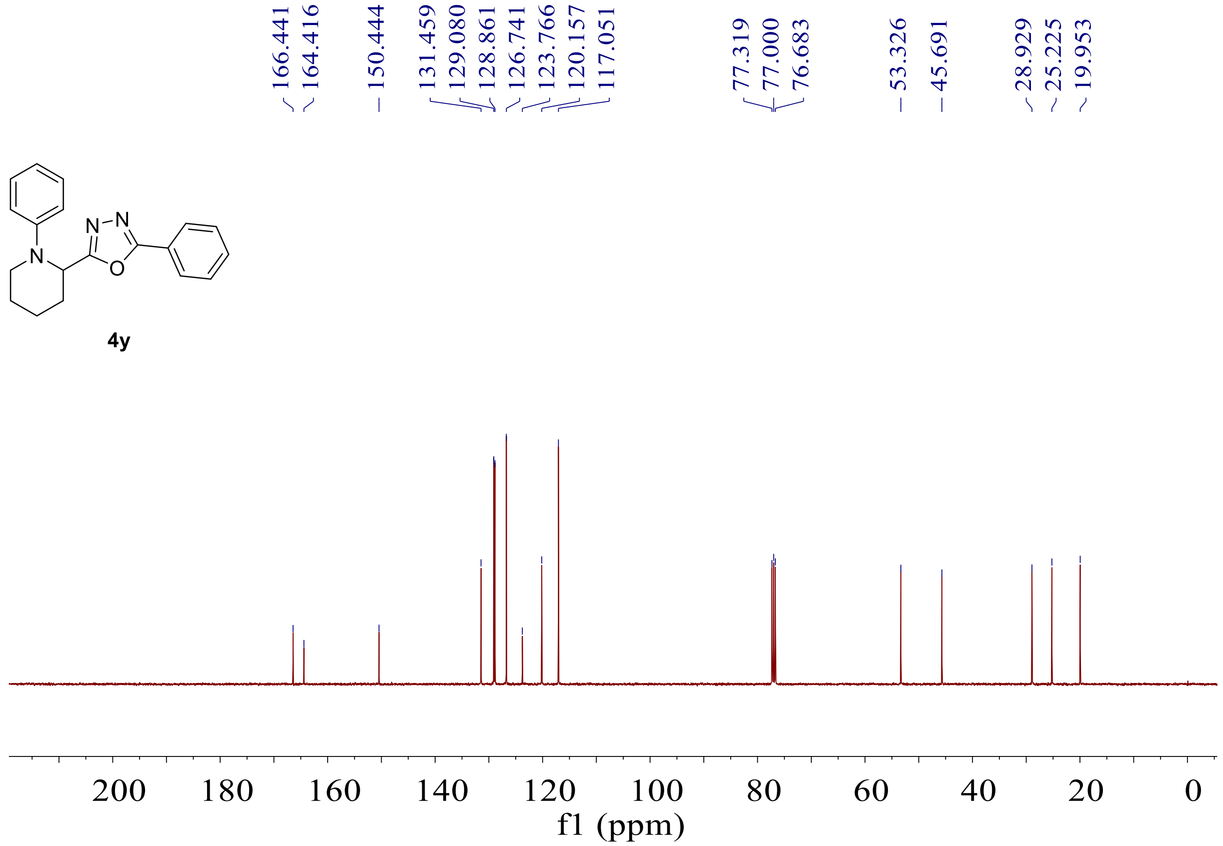

Supplement: Supplementary file 1 [file molecules-29-01253-s001.zip › molecules-2895123-supplementary.pdf]
